# Admission Liver Enzyme Elevation Grade for Risk Stratification in Critically Ill Patients: Development and Internal Validation of an Exploratory Prognostic Model

**DOI:** 10.3390/jcm15145513

**Published:** 2026-07-14

**Authors:** Giovanni Giordano, Veronica Zullino, Antonella Tosi, Giacomo Monaco, Beatrice Frasacco, Paola Celli, Franco Ruberto, Pierfrancesco Tozzi, Francesco Alessandri, Francesco Pugliese

**Affiliations:** 1 Department of General and Specialistic Surgery and Anesthesiology, “Sapienza” University of Rome, 00185 Rome, Italy; 2 Department of Emergency Care, Critical Care and Trauma, Policlinico Umberto I Hospital, 00161 Rome, Italy; v.zullino@policlinicoumberto1.it (V.Z.); g.monaco@policlinicoumberto1.it (G.M.); p.tozzi@policlinicoumberto1.it (P.T.)

**Keywords:** liver enzyme elevation, critical illness, ICU mortality, risk stratification, transaminases

## Abstract

**Background**: Liver enzyme abnormalities are common in critically ill patients, but the prognostic relevance of graded aminotransferase elevation and its timing remains uncertain. We evaluated whether Liver Enzyme Elevation (LEE) grade at ICU admission provides prognostic information beyond SAPS II in a heterogeneous ICU cohort. **Methods:** In this single-centre retrospective study, adult patients admitted to a mixed ICU between January 2023 and December 2024 and with ICU length of stay ≥72 h were analysed. LEE grade was assigned from AST and ALT according to predefined multiples of the local upper limit of normal, using the higher grade reached by either enzyme. The primary outcome was ICU mortality. Secondary outcomes included renal replacement therapy, ICU length of stay, and duration of invasive mechanical ventilation. A fixed logistic model including SAPS II and admission LEE grade was internally validated by bootstrap resampling. **Results**: Among 274 patients, ICU mortality was 27.7%. Admission LEE grade showed an adjusted association with ICU mortality (OR 1.26 per grade increase, 95% CI 1.00–1.58; *p* = 0.048), while SAPS II remained the dominant predictor (OR 1.04 per point, 95% CI 1.02–1.06; *p* < 0.001). Adding admission LEE grade to SAPS II yielded a small absolute increase in discrimination (AUC 0.737 vs. 0.703; DeLong *p* = 0.039). Bootstrap-corrected AUC was 0.730, with acceptable overall calibration. Admission LEE grade was also associated with RRT and longer ICU stay in exploratory secondary analyses. **Conclusions**: Admission LEE grade may provide modest complementary prognostic information beyond SAPS II. These findings are exploratory and require external validation before clinical implementation.

## 1. Background

Liver enzyme abnormalities are frequent and clinically relevant findings in critically ill patients and are consistently associated with increased morbidity, mortality, and risk of multiorgan failure [1,2]. In the intensive care unit (ICU), aminotransferase elevations may occur in the context of hypoperfusion, systemic inflammation, drug-related hepatotoxicity, and venous congestion, with higher rates among patients with shock, sepsis, advanced cardiac failure, or severe viral infections [3,4].

Liver-related biochemical abnormalities in this setting have been linked to adverse outcomes, including increased mortality, renal and respiratory failure, prolonged ICU length of stay, invasive mechanical ventilation, and renal replacement therapy, acting both as a marker and a potential amplifier of systemic illness [5,6,7].

Despite this clinical relevance, assessment of liver enzyme elevation in the ICU largely relies on conventional transaminases, particularly aspartate aminotransferase (AST) and alanine aminotransferase (ALT), which have important limitations [8]. These enzymes are non-specific, frequently reflect systemic rather than primary hepatic disease, and often show transient elevations that normalise after haemodynamic stabilisation [9,10]. Consequently, isolated transaminase values poorly discriminate clinically meaningful liver-related abnormalities from minor biochemical changes and are considered to be of limited prognostic value in critically ill populations [11,12].

To address these limitations, the concept of liver enzyme elevation has been proposed, aiming to classify aminotransferase abnormalities according to increasing severity rather than absolute cut-off values [13,14,15].

The Liver Enzyme Elevation (LEE) grading system is a laboratory-based classification that grades AST and ALT abnormalities according to multiples of the upper limit of normal, thereby converting continuous transaminase values into ordered severity categories [9]. This approach derives from standardized toxicity grading systems originally developed to classify adverse laboratory events in clinical trials and has subsequently been applied in infectious disease, drug-related hepatotoxicity, COVID-19, and cardiac intensive care populations. Its main advantage is simplicity and reproducibility, as it relies on routinely available laboratory tests and predefined thresholds rather than subjective clinical interpretation. However, LEE grading is not routinely incorporated into general ICU risk assessment, and its prognostic role in heterogeneous critically ill populations remains insufficiently validated.

Previous studies in selected populations have suggested an association between higher Liver Enzyme Elevation (LEE) grades and adverse outcomes [12,16]. However, systematic validation of LEE in heterogeneous ICU populations is limited, and the relative prognostic relevance of liver enzyme elevation assessed at ICU admission compared with peak values during the early ICU course remains unclear. Although preliminary evidence indicates that integrating biomarkers with established severity scores may modestly improve risk stratification [17], robust data specifically addressing transaminase-based grading systems are lacking, and the incremental prognostic value of liver enzyme kinetics beyond conventional severity scores has not been clearly established. Recent evidence has emphasized that liver enzyme abnormalities in critically ill patients frequently mirror systemic illness severity, haemodynamic instability, cardiohepatic interactions, and inflammatory burden rather than isolated hepatic disease. These findings informed the rationale of the present study by supporting the evaluation of LEE grading as a simple, reproducible, transaminase-based marker of early systemic vulnerability in a heterogeneous ICU population.

The aim of this study was to evaluate the association between liver enzyme elevation severity, assessed by the LEE grading system at ICU admission and at peak values within the first 72 h, and major ICU outcomes, including ICU mortality, length of stay, need for renal replacement therapy, and duration of invasive mechanical ventilation. For ICU mortality, we additionally aimed to determine the incremental prognostic value of admission LEE grade beyond baseline disease severity as measured by SAPS II.

## 2. Methods

### 2.1. Study Design and Population

This retrospective observational study was conducted in a mixed medical–surgical ICU of a tertiary university hospital. All consecutive adult patients (≥18 years) admitted between January 2023 and December 2024 were screened. The study was approved by the institutional ethics committee of Policlinico Umberto I (Prot. 0303/2025 approved on 26 March 2025) and conducted in accordance with the Declaration of Helsinki. Reporting followed STROBE guidelines and the TRIPOD recommendations for reporting the development and internal validation of prognostic models [18,19,20]. Patients were excluded if ICU length of stay was <72 h, if key liver biomarkers or outcome data were missing, in cases of moderate or severe traumatic liver injury (AIS-liver ≥ 2), or transfer to another institution before outcome assessment. Patients with end-stage chronic liver disease, defined as advanced or decompensated chronic liver disease with established end-stage hepatic dysfunction, were excluded. Non-end-stage chronic liver disease was not considered an exclusion criterion and was recorded as a baseline comorbidity. Therefore, the chronic liver disease variable reported in Table 1. refers to patients with documented chronic liver disease without end-stage hepatic dysfunction. 

### 2.2. Data Collection and Definitions

Demographic characteristics, comorbidities, admission diagnosis, and severity scores were extracted from electronic medical records. Disease severity was assessed using SAPS II, and comorbidity burden using the Charlson Comorbidity Index. Liver enzyme elevation was graded using the Liver Enzyme Elevation (LEE) grading system based on AST and ALT levels measured at ICU admission and at peak values within the first 72 h [21,22,23,24].

Peak liver enzyme values were assessed within the first 72 h after ICU admission. This time window was selected a priori to capture early liver enzyme elevation related to the initial critical illness insult, including haemodynamic instability, systemic inflammation, hypoxia-reperfusion injury, and early organ crosstalk, while limiting the influence of later ICU-acquired complications and time-dependent exposures. The 72 h window also allowed a standardized observation period across patients and was consistent with the study objective of evaluating the prognostic relevance of early liver enzyme abnormalities rather than delayed liver enzyme elevation occurring later during the ICU stay.

Because exclusion of patients with ICU length of stay <72 h may introduce selection and landmark bias, particularly for admission-based predictors, we performed an additional sensitivity analysis including all otherwise-eligible ICU admissions irrespective of ICU length of stay. This analysis was restricted to variables available at ICU admission and evaluated the association between admission LEE grade and ICU mortality after adjustment for SAPS II. Analyses involving peak LEE grade within 72 h were interpreted as 72 h landmark analyses and were restricted to patients with sufficient observation time for peak enzyme assessment.

LEE grade was assigned according to the highest relative elevation of AST or ALT compared with the upper limit of normal (ULN) of the reference laboratory. Concurrent bilirubin or other liver-related markers were not incorporated into the LEE grade. Total and direct bilirubin, albumin, γ-glutamyltransferase, platelet count, INR, AST/ALT ratio, and ALBI score were collected and analysed separately as descriptive or exploratory liver-related variables. In our institution, the ULN was 37 U/L for AST and 55 U/L for ALT. Patients were classified as grade 0 when both AST and ALT were <1.25 × ULN, grade 1 for values between 1.25 and 2.5 × ULN, grade 2 for values > 2.5 to 5 × ULN, grade 3 for values > 5 to 10 × ULN, and grade 4 for values > 10 × ULN. (Table 2) When AST and ALT corresponded to different grades, the highest grade was retained. LEE grade was calculated at ICU admission and again using the peak AST or ALT value recorded within the first 72 h. Because LEE grading is based on objective laboratory thresholds, its calculation is expected to be reproducible across observers; however, it remains dependent on laboratory-specific ULN values and does not identify the underlying mechanism of enzyme elevation.

Throughout the manuscript, “LEE grade” and “liver enzyme elevation” refer to the study exposure, namely graded AST/ALT elevation according to predefined ULN thresholds. LEE grade should not be interpreted as a direct measure of hepatic functional impairment or as identifying the underlying mechanism of enzyme elevation.

Hypoxic–ischemic hepatitis (HH) was defined as aminotransferase elevation ≥ 20 × ULN within 72 h in the context of circulatory, cardiac, or respiratory failure, after exclusion of alternative causes [25]. Patients with end-stage chronic liver disease were excluded. Non-end-stage chronic liver disease was not an exclusion criterion and was recorded as a baseline comorbidity. LEE grading was not adjusted for individual pre-ICU baseline transaminase values, because reliable pre-admission baseline liver enzyme levels were not systematically available. Therefore, in patients with non-end-stage chronic liver disease or chronic baseline transaminase abnormalities, LEE grade reflects the absolute degree of enzyme elevation at ICU admission or within the first 72 h rather than the change from a personal baseline.

Additional liver-related variables included bilirubin, albumin, γ-glutamyltransferase, platelet count, AST/ALT ratio, and ALBI score [26].

### 2.3. Outcomes

The primary outcome was ICU mortality. Secondary outcomes included ICU length of stay (LOS), need for renal replacement therapy (RRT), and duration of invasive mechanical ventilation.

For the primary outcome, we further evaluated the incremental prognostic performance of admission LEE grade beyond SAPS II, including discrimination, calibration, clinical utility, internal validation, and development of a prognostic nomogram.

Renal replacement therapy (RRT) was defined as new initiation of extracorporeal renal support during the ICU stay. Patients receiving chronic dialysis before ICU admission, or with chronic renal failure requiring long-term renal replacement therapy, were excluded from RRT-specific analyses. RRT initiation was based on treating physician judgement according to local ICU practice and standard clinical indications, including refractory fluid overload, severe metabolic acidosis, hyperkalaemia, uraemic complications, or persistent/worsening acute kidney injury. Detailed timing and indication-specific categories for RRT initiation were not systematically available and were therefore not included in the present analysis. ICU length of stay and duration of invasive mechanical ventilation were analysed using log-linear regression models because of their right-skewed distributions. Since death represents a competing event that may shorten observed ICU stay and ventilation duration, analyses of ICU LOS and mechanical ventilation duration were repeated and primarily interpreted in ICU survivors. Additional sensitivity analyses, including analyses excluding patients with hypoxic–ischemic hepatitis, are reported in the Supplementary Material.

### 2.4. Statistical Analysis

Continuous variables are reported as medians with interquartile ranges (IQR), and categorical variables as counts and percentages. Group comparisons were performed using the Mann–Whitney U test or χ^2^/Fisher’s exact test, as appropriate. Associations with ICU mortality were explored using logistic regression. For the primary mortality analysis, the prognostic model was specified a priori to evaluate the incremental prognostic value of admission LEE grade beyond SAPS II. Therefore, the primary model included SAPS II and admission LEE grade as fixed predictors, both entered as one-degree-of-freedom terms. This model was chosen to directly address the main study objective and was not derived through automated, stepwise, or performance-driven variable selection. Adjusted marginal effects were used to visualize predicted mortality across LEE grades and SAPS II strata.

For mortality model development, candidate variables considered for exploratory and sensitivity analyses were selected based on clinical plausibility, availability at or shortly after ICU admission, previous evidence of association with ICU outcomes, and univariate association with ICU mortality [27,28].

Variables considered included baseline severity of illness, demographic characteristics, comorbidity burden, selected comorbidities, admission diagnosis, and liver-related markers, including admission LEE grade, peak LEE grade within 72 h, and hypoxic–ischemic hepatitis. Automated stepwise variable selection was not used. Instead, additional clinically motivated models were evaluated as exploratory and sensitivity analyses to assess robustness, collinearity, timing of LEE assessment, and alternative variable specifications, rather than to select the model with the best apparent performance. In particular, admission and peak LEE grades were assessed in separate models to avoid collinearity and to preserve clinical interpretability.

The events-per-variable ratio was formally considered during model development. Given 76 ICU deaths, the fixed primary model including SAPS II and admission LEE grade provided approximately 38 events per predictor variable. More extended exploratory models were kept parsimonious and maintained an events-per-variable ratio of approximately 15 or higher. Interaction and nonlinearity were also explored. The interaction between SAPS II and admission LEE grade was assessed in an exploratory model but was not retained in the primary model in order to preserve parsimony, avoid overfitting, and maintain bedside interpretability. Nonlinear associations were investigated by comparing ordinal and categorical specifications of LEE grade and by assessing nonlinear modelling of SAPS II; these exploratory analyses did not materially change the interpretation of the primary model. Accordingly, the primary model was retained as a fixed, clinically prespecified model designed to evaluate the incremental prognostic value of admission LEE grade beyond SAPS II, rather than to maximize in-sample predictive performance.

Model discrimination was assessed using receiver operating characteristic (ROC) curves and area under the curve (AUC). Ninety-five percent confidence intervals for AUC estimates were calculated using DeLong’s method, and incremental prognostic value was evaluated by comparing correlated ROC curves using DeLong’s test. Clinical usefulness was assessed using decision curve analysis across a range of threshold probabilities, according to established methodological recommendations [29,30].

In this context, threshold probabilities were interpreted as the predicted mortality risk at which a clinician would consider a patient sufficiently high-risk to justify intensified monitoring or additional risk-stratification efforts.

Internal validation of the fixed primary mortality model was performed using bootstrap resampling with 1000 iterations to estimate optimism-corrected performance. Because the primary model was specified a priori and no automated or performance-driven model selection algorithm was used to define it, model selection was not incorporated into the bootstrap loop. Model calibration was assessed both graphically and quantitatively. Calibration-in-the-large and overall calibration were evaluated using the calibration intercept and calibration slope, respectively, obtained from a logistic calibration model regressing the observed outcome on the model linear predictor. Overall prediction error was quantified using the Brier score, calculated as the mean squared difference between predicted probabilities and observed outcomes. Apparent and bootstrap optimism-corrected estimates were reported for the primary mortality model.

Based on the internally validated fixed primary model, a nomogram was developed to facilitate individualized estimation of ICU mortality risk. The nomogram was considered exploratory and hypothesis-generating, pending external validation. Model development, internal validation, and performance reporting were conducted in line with TRIPOD guidance.

Given the heterogeneity of the cohort and the significant univariate association between admission diagnosis and ICU mortality, we performed additional sensitivity analyses to assess whether the association between admission LEE grade and ICU mortality was influenced by the large trauma subgroup. Because several admission diagnosis categories included a small number of patients, admission diagnosis was not entered as a full multi-category variable in the primary model. Instead, trauma status was modelled as a binary variable, comparing trauma versus non-trauma admissions. The primary mortality model was therefore refitted after adding trauma status to SAPS II and admission LEE grade. In addition, the SAPS II plus admission LEE grade model was repeated after excluding trauma patients. An exploratory interaction between admission LEE grade and trauma status was also assessed.

Renal replacement therapy (RRT) was defined as new initiation of extracorporeal renal support during the ICU stay. Patients receiving chronic dialysis before ICU admission, or with chronic renal failure requiring long-term renal replacement therapy, were excluded from RRT-specific analyses. RRT initiation was based on treating physician judgement according to local ICU practice and standard clinical indications, including refractory fluid overload, severe metabolic acidosis, hyperkalaemia, uraemic complications, or persistent/worsening acute kidney injury. Detailed timing and indication-specific categories for RRT initiation were not systematically available and were therefore not included in the present analysis.

ICU LOS and duration of mechanical ventilation were analyzed using log-linear regression models to account for right-skewed distributions. Since death represents a competing event that may shorten observed ICU stay and ventilation duration, analyses of ICU LOS and mechanical ventilation duration were repeated and primarily interpreted in ICU survivors. Additional sensitivity analyses, including analyses excluding patients with hypoxic–ischemic hepatitis, are reported in the Supplementary Material. Associations between LEE grade and RRT were evaluated using logistic and ordinal regression models after excluding patients with chronic renal failure.

Detailed univariate analyses, alternative multivariable specifications, sensitivity analyses, and additional exploratory analyses for all study outcomes are reported in the Supplementary Material.

All tests were two-sided, with *p* < 0.05 considered statistically significant.

The extent of missing data was assessed for all variables included in the descriptive and inferential analyses and is reported in Supplementary Table S1. Missingness was generally low in the final study cohort. Variables included in the primary prognostic model, namely SAPS II, admission LEE grade, and ICU mortality, were complete, as were the key liver biomarkers required for LEE grading. Most variables had less than 5% missing data; total proteins was the only variable exceeding this threshold and was used only in descriptive or exploratory analyses, not in the primary prognostic model. Given the low proportion of missing data in clinically relevant variables, the absence of missingness in the primary outcome and key predictors, and the parsimonious structure of the prognostic models, complete-case analysis was considered appropriate. Multiple imputation was not performed because it would have introduced additional modelling assumptions with limited expected benefit in the context of very limited missingness for the main analytical variables.

Statistical analyses were performed using R version 4.5.2 (R Foundation for Statistical Computing, Vienna, Austria). Logistic regression models were fitted using base R functions. ROC analyses, AUC estimates, 95% confidence intervals, and DeLong tests were performed using the pROC package (v1.19.0.1). Bootstrap internal validation was performed using the boot package (v1.3.32), while nomogram development and calibration analyses were performed using the rms package (v8.1.0). Decision curve analysis was performed using the rmda package (v1.6). Data management and visualization were performed using readxl (v1.4.5), dplyr (v1.2.0), and ggplot2 (v4.0.1).

## 3. Results

**Figure 1:**
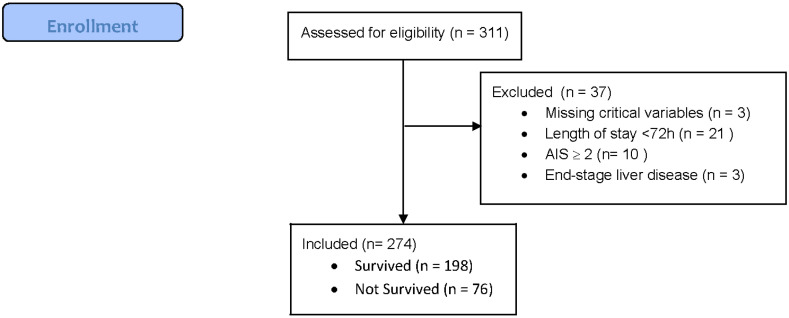
Flow diagram illustrating patient screening, exclusion criteria with the corresponding number of excluded patients for each criterion, and final inclusion in the study cohort.

**Figure 2:**
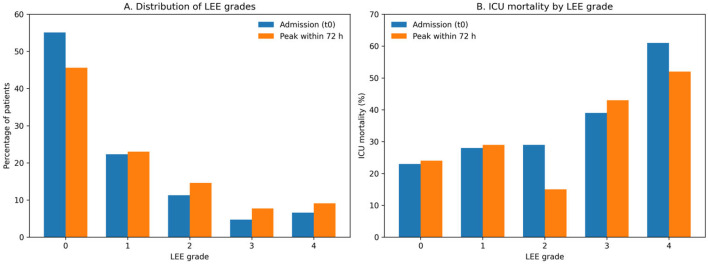
Distribution of liver enzyme elevation (LEE) grades and associated ICU mortality. (**A**) Distribution of LEE grades at ICU admission (t0) and peak values within the first 72 h. (**B**) Crude ICU mortality stratified by LEE grade at ICU admission and by peak LEE grade within 72 h.

Of the 274 patients included, 198 (72.3%) were survivors and 76 (27.7%) non-survivors [Figure 1]. Baseline characteristics are reported in Table 1. The median age was 60 years (IQR 47–73), with a median SAPS II score of 41.5 (IQR 33.25–55). Respiratory conditions were the leading cause of ICU admission, followed by trauma, neurological, cardiovascular, and sepsis/abdominal presentations. More than half of the cohort had LEE grade 0 and 6.6% had LEE grade 4; peak values within 72 h increased the proportion of LEE grade 4 to 9.1% [Figure 2A]. Overall, ICU mortality was 27.7%, median ICU length of stay (LOS) was 14.5 days (IQR 7–26), median duration of invasive mechanical ventilation was 10 days (IQR 4–20), and renal replacement therapy (RRT) was required in 15.7% [Table 3].

### 3.1. ICU Mortality

Survivors and non-survivors differed significantly in age, baseline disease severity, and comorbidity burden [Table 4]. Univariate associations with ICU mortality are reported in Supplementary Table S2. Liver enzyme elevation severity was greater among non-survivors, with significant differences in both admission and peak LEE grade distributions. ICU mortality across LEE grades is reported in Figure 2B and Supplementary Figure S1.

Consistent with the prespecified objective of evaluating the incremental prognostic value of admission LEE grade beyond SAPS II, the primary mortality model included SAPS II and admission LEE grade. Additional models assessing peak LEE grade and alternative specifications are reported as exploratory and sensitivity analyses [Supplementary Tables S3–S5; Supplementary Figures S2 and S3].

**Figure 3:**
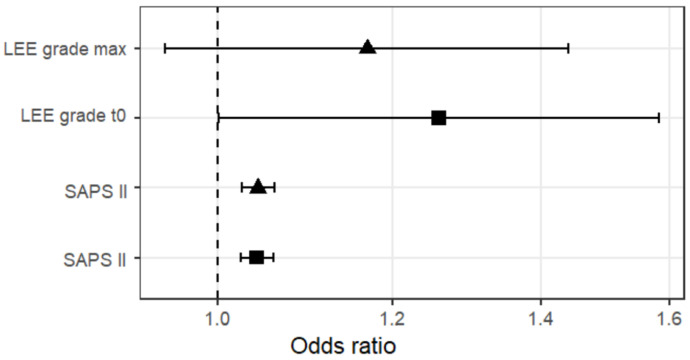
Forest plot of multivariable logistic regression models for ICU mortality. Forest plot showing adjusted odds ratios (ORs) and 95% confidence intervals for ICU mortality derived from two separate multivariable logistic regression models. Squares represent the model including SAPS II and LEE grade at ICU admission (t0), whereas triangles represent the model including SAPS II and maximum LEE grade during the ICU stay. SAPS II estimates from the two models are displayed on separate rows. Squares represent estimates from the model including LEE grade at admission, whereas triangles represent estimates from the model including maximum LEE grade. All estimates are adjusted within their respective models.

The primary mortality model was defined as follows: logit(p) = −3.067 + 0.0409 × SAPS II + 0.2299 × admission LEE grade, where p represents the predicted probability of ICU mortality (Figure 3). Both SAPS II (OR 1.04 per point, 95% CI 1.02–1.06; *p* < 0.001) and admission LEE grade (OR 1.26 per grade increase, 95% CI 1.00–1.58; *p* = 0.048) were independently associated with ICU mortality [Table 5].

**Figure 4:**
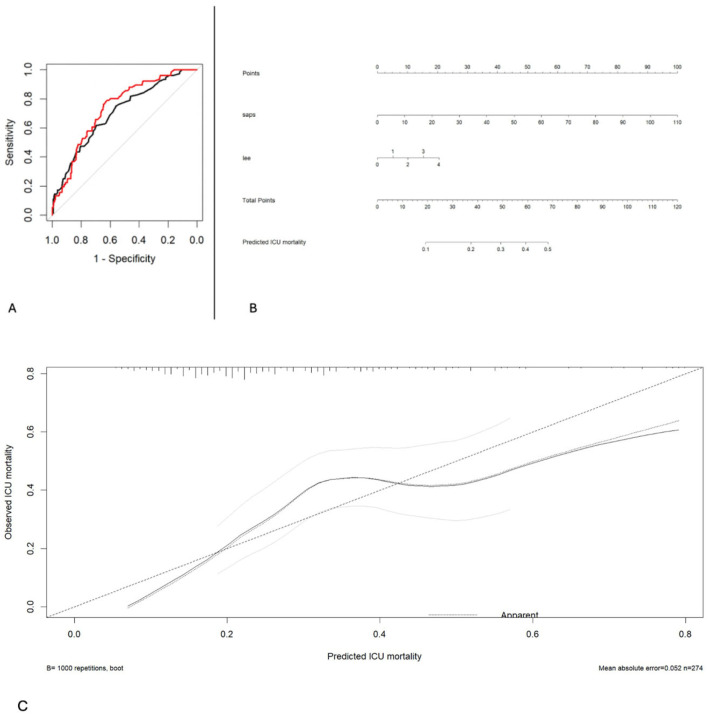
Discrimination, clinical applicability, and calibration of the ICU mortality prediction model. (**A**) Receiver operating characteristic (ROC) curves comparing the discriminative performance of the model including SAPS II alone (black line) and the combined model including SAPS II plus admission liver enzyme elevation (LEE) grade (red line) for the prediction of ICU mortality. The addition of admission LEE grade was associated with a small absolute increase in AUC compared with SAPS II alone (AUC 0.737, 95% CI 0.674–0.799 vs. AUC 0.703, 95% CI 0.635–0.771; DeLong test *p* = 0.039). (**B**) Nomogram derived from the final multivariable model incorporating SAPS II and admission LEE grade, developed to facilitate individualized ICU mortality risk estimation. (**C**) Bootstrap-corrected calibration plot of the final SAPS II plus admission LEE grade model, showing the agreement between predicted and observed ICU mortality. The diagonal line represents perfect calibration, while the solid and dotted curves indicate apparent and bias-corrected calibration, respectively, after 1000 bootstrap resamples. Quantitative calibration metrics are reported in  Supplementary Table S7. Visual inspection suggests acceptable overall calibration, with some deviation from the ideal diagonal in the mid-to-high predicted risk range.

In ROC analysis, SAPS II alone showed fair discrimination for ICU mortality, with an AUC of 0.703 (95% CI 0.635–0.771). Incorporation of admission LEE grade was associated with a small absolute increase in discrimination, with an AUC of 0.737 (95% CI 0.674–0.799; DeLong *p* = 0.039) [Figure 4A].

At the optimal cut-off probability (0.229), the combined model demonstrated high sensitivity (78.9%) and negative predictive value (88%), with modest specificity (62.1%) and positive predictive value (44%) [Supplementary Table S6].

Model discrimination, clinical applicability, and calibration are summarised in Figure 4. Based on the fixed primary multivariable model, a nomogram was developed to facilitate individualized ICU mortality risk estimation. Quantitative performance metrics supported acceptable overall calibration. For the primary SAPS II plus admission LEE grade model, the apparent AUC was 0.737, the calibration intercept was 0.000, the calibration slope was 1.000, and the Brier score was 0.176. After bootstrap optimism correction, the model showed an AUC of 0.730, a calibration intercept of −0.010, a calibration slope of 0.975, and a Brier score of 0.181 [Supplementary Table S7]. Although these metrics suggested acceptable overall calibration and limited evidence of overfitting, visual inspection of the bootstrap-corrected calibration curve showed some deviation from the ideal diagonal in the mid-to-high predicted risk range, approximately between 0.3 and 0.6. This finding suggests possible local miscalibration in this risk interval and should be interpreted cautiously given the modest sample size and limited number of patients at higher predicted risk.

Decision curve analysis demonstrated a higher net clinical benefit for the combined SAPS II plus admission LEE model compared with SAPS II alone across clinically relevant threshold probabilities (approximately 10–20%), with both models outperforming treat-all and treat-none strategies [Supplementary Figure S4].

To address the potential impact of excluding patients with ICU length of stay shorter than 72 h, we performed an additional admission-based sensitivity analysis including all otherwise-eligible ICU admissions, irrespective of ICU length of stay. This expanded cohort included 295 patients, of whom 88 (29.8%) died in the ICU. The 21 patients with ICU length of stay <72 h included 12 ICU deaths and 9 patients discharged alive from the ICU before 72 h. Supplementary Table S8. In this sensitivity analysis, admission LEE grade remained independently associated with ICU mortality after adjustment for SAPS II (OR 1.306 per grade increase, 95% CI 1.046–1.629; *p* = 0.018). Supplementary Table S9. SAPS II alone showed an AUC of 0.740 (95% CI 0.679–0.802), whereas the SAPS II plus admission LEE grade model showed an AUC of 0.770 (95% CI 0.714–0.826; DeLong *p* = 0.038). Supplementary Table S10. These findings were consistent with the primary 72 h cohort analysis and support the robustness of the admission-based association between LEE grade and ICU mortality.

Trauma patients represented a substantial subgroup of the cohort and had lower crude ICU mortality than non-trauma patients. In the 72 h cohort, trauma patients accounted for 65 of 274 patients and had an ICU mortality rate of 15.4%, compared with 31.6% among non-trauma patients. To assess whether this diagnostic heterogeneity influenced the primary findings, we performed additional trauma-focused sensitivity analyses.

When trauma status was added to the primary mortality model, admission LEE grade remained associated with ICU mortality after adjustment for SAPS II and trauma status (OR 1.280 per grade increase, 95% CI 1.017–1.611; *p* = 0.035). Trauma admission was associated with lower adjusted odds of ICU mortality, although this did not reach conventional statistical significance (OR 0.501, 95% CI 0.233–1.080; *p* = 0.078). In a non-trauma-only analysis, the association between admission LEE grade and ICU mortality was attenuated and was no longer statistically significant (OR 1.181, 95% CI 0.920–1.515; *p* = 0.192). An exploratory interaction analysis showed no clear evidence that the association between admission LEE grade and mortality differed between trauma and non-trauma patients, although this analysis was underpowered (LEE grade × trauma interaction OR 1.672, 95% CI 0.816–3.427; *p* = 0.161). These findings are reported in Supplementary Table S11.

### 3.2. Secondary Outcomes

Secondary outcomes included renal replacement therapy, length of stay, and invasive mechanical ventilation.

RRT analyses were performed after excluding patients with chronic renal failure or chronic dialysis at baseline, thereby focusing on new RRT requirement during the ICU stay. In this restricted cohort, both admission and peak LEE grades were associated with RRT requirement, and the association remained robust after adjustment for SAPS II and metabolic covariates [Supplementary Tables S12–S15].

**Figure 5:**
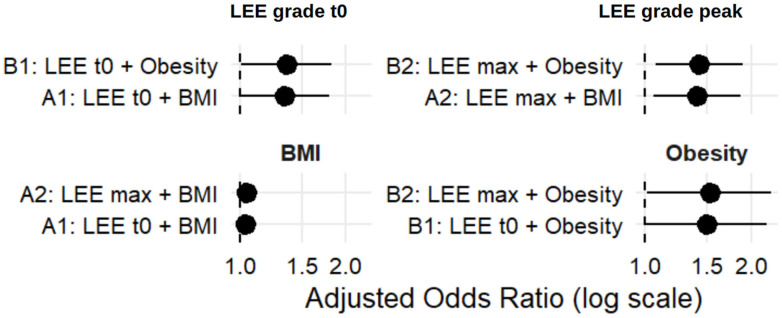
Reduced multivariable forest plot for the association of LEE severity and body habitus with the need for dialysis. Forest plot showing adjusted odds ratios (ORs) and 95% confidence intervals for LEE grade at admission (t0), peak LEE grade within 72 h, BMI, and obesity across four multivariable logistic regression models. Models A1 and A2 include BMI as a continuous variable, whereas Models B1 and B2 incorporate obesity as a categorical predictor. In each model, LEE severity was evaluated either at admission (t0) or at peak (max). All models were adjusted for SAPS II and diabetes. The figure highlights the robustness of the association between LEE severity and dialysis across modeling strategies, and the consistent effect of obesity when modeled categorically.

RRT rates rose markedly across LEE grades, particularly at grade 4, as shown in Supplementary Figure S5. Linearity testing supported modeling LEE grade as an ordinal variable, with no evidence of deviation from linearity for either admission or peak values [Supplementary Table S13]. In multivariable models [Figure 5, Supplementary Table S14], LEE severity remained an independent determinant of RRT, with adjusted ORs between 1.34 and 1.43 per grade. The association between LEE severity and dialysis requirement remained robust after minimal adjustment for overall illness severity using SAPS II [Supplementary Table S15].

Boxplots illustrating ICU length of stay across admission and peak LEE grades are shown in Supplementary Figures S6 and S7. Because death may act as a competing event for ICU length of stay and duration of invasive mechanical ventilation, these outcomes were analysed both in the overall cohort and in ICU survivors, with survivor-restricted analyses considered the primary interpretation for these secondary outcomes. In the overall cohort, admission LEE grade showed a stronger association with ICU length of stay than peak LEE grade [Supplementary Table S16]. This pattern was confirmed after adjustment for SAPS II, with admission LEE grade remaining independently associated with longer ICU stay, whereas the association with peak LEE grade was weaker and did not reach conventional statistical significance [Supplementary Tables S17 and S18].

**Figure 6:**
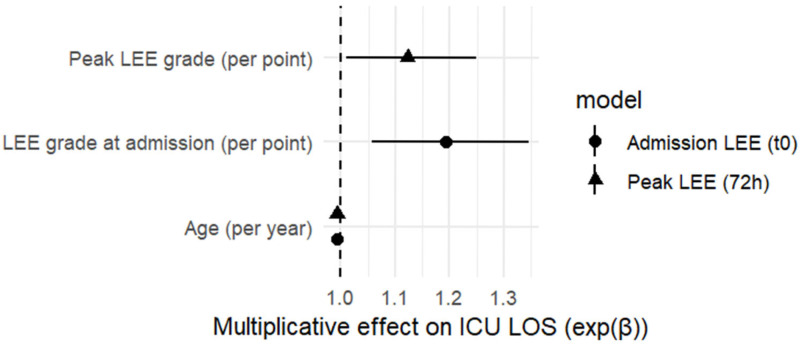
Effect of admission versus peak LEE grade on ICU length of stay in patients without hypoxic hepatitis. Forest plot showing the multiplicative effects (exp(β)) of LEE grade at ICU admission and peak LEE grade at 72 h on ICU length of stay among patients without hypoxic hepatitis. Estimates are expressed per one-point increase in LEE grade and adjusted for age. Points represent point estimates, horizontal bars indicate 95% confidence intervals, and the dashed vertical line denotes the null effect (exp(β) = 1).

Among ICU survivors, both admission and peak LEE grades were associated with longer ICU length of stay in univariate analyses [Supplementary Table S19]. In multivariable survivor-restricted models adjusted for age, admission LEE grade remained independently associated with longer ICU stay [Figure 6; Supplementary Table S20], while peak LEE grade showed a weaker association [Supplementary Table S21]. In sensitivity analyses excluding patients with hypoxic–ischemic hepatitis, the association persisted for admission LEE grade but was attenuated for peak LEE grade, further supporting the more consistent prognostic relevance of admission LEE [Supplementary Tables S22 and S23; Figure 6]. Marginal-effects visualisations demonstrated a stepwise increase in predicted ICU length of stay with higher LEE grades, with a steeper gradient for admission LEE compared with peak LEE within 72 h [Supplementary Figure S8]. Finally, multivariable analyses using log-transformed AST and ALT values showed that admission AST, but not admission ALT, was associated with ICU length of stay among survivors, whereas peak AST and ALT values within 72 h were not independently associated with ICU stay duration [Supplementary Tables S24 and S25].

Duration of invasive mechanical ventilation was analysed among ICU survivors requiring ventilatory support, to reduce distortion related to death as a competing event. Based on univariate screening analyses, variables showing potential associations with ventilation duration were entered into multivariable log-linear models [Supplementary Table S26]. In the main multivariable model, obesity was the strongest determinant of prolonged ventilation, while admission LEE grade remained independently associated with longer ventilation duration, corresponding to an estimated 22% increase per one-grade increase in LEE severity [Supplementary Table S27]. Additional robustness analyses explored alternative representations of adiposity, including BMI as a continuous variable and obesity class, as well as different LEE timing definitions. These analyses confirmed the dominant association of adiposity with ventilation duration and showed that the association was more consistent for admission LEE grade than for peak LEE grade within 72 h [Supplementary Tables S28–S30].

## 4. Discussion

In this heterogeneous ICU cohort, liver enzyme elevation severity assessed by LEE grade at ICU admission showed adjusted associations with ICU mortality and other clinically relevant outcomes, including renal replacement therapy, ICU length of stay, and duration of mechanical ventilation.

A relevant observation of this study is the different behaviour of admission and peak LEE grades. Admission LEE grade showed more consistent associations with adverse outcomes than peak LEE grade, particularly for ICU mortality and duration of mechanical ventilation. This finding may be biologically plausible, because early transaminase elevation could reflect the severity of the initial haemodynamic and inflammatory insult, hypoxia-reperfusion injury, and early microcirculatory dysfunction [10,31]. However, this interpretation should be considered hypothesis-generating rather than mechanistically demonstrated. The comparison between admission and peak LEE grades is inherently affected by the study design, including the exclusion of patients with ICU length of stay shorter than 72 h and the fact that peak enzyme values are susceptible to time-dependent treatment effects, evolving clinical course, fluid resuscitation, vasopressor exposure, procedures, transfusions, drug exposure, and delayed ICU-acquired complications. Therefore, the apparently stronger prognostic signal of admission LEE grade should not be interpreted as definitive evidence that admission liver enzyme elevation is biologically superior to peak liver enzyme elevation. Rather, admission LEE grade may represent a simpler and temporally cleaner marker of early systemic vulnerability, whereas peak LEE grade should be interpreted within the constraints of a 72 h landmark analysis.

The choice of a 72 h window for peak LEE assessment was intended to balance biological plausibility and methodological standardization. It was considered sufficiently long to capture early enzyme trajectories after the initial critical illness insult, but sufficiently short to reduce confounding from delayed ICU-related exposures, such as secondary infections, drug-induced hepatotoxicity, transfusions, procedures, or prolonged haemodynamic support.

Importantly, these processes are not confined to the liver but mirror global organ dysfunction, a pattern repeatedly linked to poor outcomes in acute heart failure, sepsis, and severe COVID-19 [5,27,28,29]. Consequently, early identification of transaminase abnormalities may help clinicians recognize patients with greater systemic vulnerability and higher risk of adverse outcomes [32]. However, given the retrospective observational design of the present study, our findings do not establish that LEE-guided interventions improve clinical outcomes. Rather, LEE grading should be interpreted as a potential risk-stratification marker that may prompt closer clinical attention and generate hypotheses for prospective studies evaluating whether targeted optimization strategies can modify patient-centred outcomes [10].

The persistence of associations between admission LEE grade and ICU length of stay among survivors supports the prognostic relevance of early liver enzyme elevation beyond imminent death. Peak values, conversely, may be influenced by time-dependent exposures and evolving clinical course, diluting their prognostic signal [33,34,35,36]. However, because aminotransferase elevations in the ICU may arise from multiple overlapping mechanisms, LEE grade should not be interpreted as identifying a specific hepatic injury pathway. Rather, it likely integrates the effects of haemodynamic instability, systemic inflammation, venous congestion, hypoxic–ischemic injury, drug exposure, and underlying liver vulnerability. Survivorship bias may also influence these associations, as patients who die early may not reach extreme enzyme elevations, whereas those with longer survival may exhibit higher peaks due to ongoing injury or delayed recovery [37].

Severity scores at ICU admission, such as APACHE and SAPS, show robust discrimination and calibration for ICU mortality prediction, frequently exceeding the performance of individual biomarkers or isolated laboratory measures [38,39,40]. Studies assessing the integration of serial biomarker measurements into established severity scores have reported modest incremental gains in mortality prediction, particularly in patients with acute inflammatory or septic conditions [17,41,42]. However, the added prognostic value is biomarker-specific and often limited, with small and population-dependent increases in AUROC. The LiFe score represents one of the most directly comparable liver-specific prognostic tools developed for critically ill patients [13]. However, LiFe and LEE capture different biological domains and were designed for different clinical contexts. The LiFe score was developed primarily to predict outcome in ICU patients with chronic liver disease or acute-on-chronic liver failure and is based on arterial lactate, total bilirubin, and INR at ICU admission, thereby integrating markers of tissue hypoperfusion, cholestatic/excretory dysfunction, and hepatic synthetic dysfunction. In contrast, the LEE grading system used in the present study is based exclusively on AST and ALT elevations relative to the upper limit of normal and was intended to provide a simple, transaminase-based measure of early hepatocellular injury severity in a heterogeneous ICU population without end-stage chronic liver disease. Therefore, LEE should not be considered a replacement for LiFe, but rather a different and complementary approach focused on early aminotransferase abnormalities. A formal head-to-head comparison with the LiFe score could not be performed in the present cohort because arterial lactate at ICU admission was not systematically available in the study dataset. Although total bilirubin and INR were collected, deriving a modified LiFe score without lactate would not reproduce the original score and could generate misleading results. Accordingly, the present findings should be interpreted as supporting the exploratory prognostic relevance of admission transaminase-based LEE grading, rather than demonstrating superiority over established liver-specific prognostic scores. Future studies should directly compare LEE, LiFe, MELD-based indices, and general ICU severity scores in externally validated cohorts.

In our study, age and SAPS II remained the dominant independent predictors for ICU mortality in fully adjusted models. However, in a parsimonious model in which age was excluded because of a high risk of multicollinearity with SAPS II, admission LEE grade was independently associated with mortality and modestly improved discrimination when added to SAPS II.

Although the addition of admission LEE grade to SAPS II was associated with a statistically significant improvement in discrimination, the absolute increase in AUC was small and the incremental prognostic value of admission LEE grade should therefore be interpreted cautiously. The adjusted association with ICU mortality was at the margin of conventional statistical significance, and no additional reclassification metrics such as NRI or IDI were calculated. Accordingly, these findings should be viewed as supportive and hypothesis-generating rather than as definitive evidence of clinically meaningful improvement in mortality prediction, and they should not be interpreted as suggesting that LEE grade can replace established ICU severity scores. Rather, admission LEE grade may provide modest complementary prognostic information, potentially reflecting early systemic injury and physiological vulnerability. Decision curve analysis suggested a higher net benefit for the combined model mainly across lower threshold probabilities, corresponding to settings in which clinicians prioritize early identification of patients at increased risk rather than highly specific prediction of death. However, the magnitude of improvement remains insufficient to support routine implementation of the model or nomogram as standalone ICU risk assessment tools. Based on the internally validated model, we developed a nomogram as a pragmatic aid to support individualized risk estimation. Although quantitative calibration metrics suggested acceptable overall calibration and bootstrap internal validation showed limited optimism, the bootstrap-corrected calibration curve showed some deviation from the ideal diagonal in the mid-to-high predicted risk range. This may reflect local miscalibration related to the modest sample size, the limited number of events, and the relatively small number of patients with high predicted mortality. Therefore, given the modest incremental discrimination, possible local miscalibration, and absence of external validation, individual risk estimates derived from the nomogram should be interpreted cautiously, particularly in higher-risk patients, and the nomogram should currently be regarded as an exploratory supportive tool pending external validation in independent cohorts.

Transaminase elevation is commonly associated with acute kidney injury in critical illness, reflecting a shared systemic hypoxic–ischemic insult, and the coexistence of hepatic dysfunction and AKI confers a worse prognosis [43,44]. In patients requiring renal replacement therapy, concurrent liver dysfunction at initiation is independently associated with higher ICU mortality, particularly in the presence of oligoanuria, acidosis, and multiorgan failure. In our cohort, the association between LEE severity and renal replacement therapy was robust and dose dependent. These findings support shared pathophysiological pathways linking hepatic and renal dysfunction in critical illness, including hypoperfusion, venous congestion, inflammation, and microcirculatory impairment [45,46].

In critically ill patients, elevations of aminotransferases (AST and/or ALT) at ICU admission are associated with greater disease severity, longer ICU length of stay and need of invasive mechanical ventilation, particularly when increases are pronounced or sustained [10,35,38,47,48]. In our study, admission LEE grade was also consistently associated with ICU length of stay and prolonged mechanical ventilation, independent of disease severity and evident among survivors, as analyses were restricted to this group to avoid distortion of length-of-stay estimates by early mortality. Similar associations after liver transplantation suggest that ongoing hepatic injury may hinder respiratory recovery [49]. Together, these findings suggest that early liver enzyme elevation may act as a marker of physiological reserve and systemic stress, identifying patients more likely to require prolonged critical care.

This study has several limitations that warrant consideration. First, its retrospective, single-centre design limits the generalisability of the findings and precludes any definitive causal inference. The associations observed should therefore be interpreted as prognostic and hypothesis-generating rather than causal. In addition, the relatively modest sample size limited statistical power for detailed subgroup analyses and required the use of parsimonious models. Although bootstrap internal validation suggested acceptable optimism-corrected discrimination and calibration, internal validation cannot substitute for independent external validation. Case-mix, admission patterns, severity distribution, resuscitation practices, and laboratory measurement protocols may differ substantially across centres, potentially affecting model calibration, discrimination, and clinical utility. Accordingly, the nomogram should currently be regarded as an internally validated exploratory tool rather than a model ready for widespread clinical implementation.

Second, although multivariable adjustment was performed, the potential for residual confounding remains substantial. Liver enzyme elevation in critically ill patients is a non-specific finding and may result from several overlapping mechanisms, including sepsis, circulatory shock, cardiac dysfunction and venous congestion, hypoxic–ischemic injury, drug-induced hepatotoxicity, transfusion burden, and pre-existing liver disease. Several potentially relevant variables, including detailed haemodynamic parameters, vasopressor dose and duration, transfusion requirements, and exposure to potentially hepatotoxic medications, were not incorporated into the primary models. These factors may have influenced both LEE grade and clinical outcomes. Moreover, some of these variables may act not only as confounders but also as mediators or markers of the same systemic injury process linking critical illness severity, liver enzyme elevation, and mortality. Therefore, admission LEE grade should be interpreted as an integrated prognostic marker of systemic physiological stress rather than as a liver-specific determinant of adverse outcome.

Third, the exclusion of patients with ICU length of stay shorter than 72 h represents an important methodological limitation. This criterion was used to allow standardized assessment of peak liver enzyme elevations within the predefined 72 h window; however, it may have introduced selection and landmark bias by excluding patients with fulminant early trajectories, including early deaths, as well as patients discharged alive before 72 h. To address this issue for admission-based predictors, we performed a sensitivity analysis including all otherwise-eligible ICU admissions irrespective of ICU length of stay, which yielded findings consistent with the primary analysis. Nevertheless, analyses involving peak LEE grade within 72 h should be interpreted as 72 h landmark analyses and may not be generalizable to patients who die or are discharged before completion of the 72 h observation window. Furthermore, peak liver enzyme values remain susceptible to time-dependent confounding related to evolving clinical course and ICU interventions, including fluid resuscitation, vasopressor exposure, procedures, transfusions, and drug exposure.

Fourth, because pre-admission baseline liver enzyme values were not systematically available, LEE grades were assigned using absolute AST and ALT elevations relative to the local laboratory upper limit of normal rather than changes from individual baseline values. Consequently, in patients with non-end-stage chronic liver disease or chronic baseline transaminase abnormalities, some degree of misclassification cannot be excluded. Moreover, although LEE grading is simple and based on reproducible laboratory thresholds, its application remains dependent on laboratory-specific reference ranges and does not identify the underlying mechanism of enzyme elevation.

Fifth, a formal comparison with the LiFe score could not be performed because arterial lactate at ICU admission was not systematically available. Since lactate is an essential component of the original LiFe score, calculating a modified version based only on bilirubin and INR would not have been methodologically appropriate. Future studies should include the variables required to calculate LiFe and directly compare its prognostic performance with LEE grading, MELD-based indices, and general ICU severity scores in both heterogeneous ICU populations and liver-enriched subgroups.

Sixth, the cohort was clinically heterogeneous, and trauma patients represented a substantial subgroup with lower crude mortality than other admission categories. Although sensitivity analyses accounting for trauma status and excluding trauma patients were performed, the study was not powered for robust diagnostic subgroup modelling. The attenuation of the LEE effect in the non-trauma-only analysis suggests that diagnostic case-mix may influence the apparent prognostic contribution of admission LEE grade. Therefore, the prognostic relevance of LEE grading should be externally validated both in heterogeneous ICU cohorts and in more homogeneous diagnostic subgroups.

Finally, LEE grading is not yet a routinely implemented tool in general critical care practice. Prior applications have mainly involved selected populations, including patients with drug-related hepatotoxicity, infectious diseases, COVID-19, or cardiac critical illness, and its performance may vary according to case-mix, baseline liver disease prevalence, laboratory reference ranges, and the dominant mechanisms of enzyme elevation. Therefore, our findings should be regarded as an additional step toward validation of LEE grading in heterogeneous ICU populations rather than definitive evidence supporting its broad implementation. Prospective multicentre studies are warranted to externally validate admission LEE grading, further assess calibration and clinical utility, and determine whether LEE-informed risk stratification can modify clinical decisions or improve patient-centred outcomes.

## 5. Conclusions

In this single-centre cohort of critically ill patients surviving at least 72 h after ICU admission, liver enzyme elevation severity assessed by LEE grade at ICU admission showed an adjusted association with ICU mortality and other clinically relevant outcomes, including renal replacement therapy, ICU length of stay, and duration of mechanical ventilation. The admission-based association between LEE grade and ICU mortality was further supported by a sensitivity analysis including all otherwise-eligible ICU admissions irrespective of ICU length of stay. Admission LEE grade provided modest complementary prognostic information beyond the established SAPS II score, whereas peak liver enzyme elevations within 72 h were less consistently informative. However, the main LEE effect was borderline, the absolute gain in discrimination was small, and the clinical relevance of this improvement should therefore be interpreted cautiously. These findings, supported by acceptable calibration and bootstrap internal validation, are compatible with the hypothesis that early liver enzyme abnormalities may act as markers of systemic vulnerability and occult physiological stress rather than isolated hepatic dysfunction; however, this mechanistic interpretation was not directly tested and should be considered hypothesis-generating.

The derived nomogram may provide a pragmatic framework for early, individualized risk stratification; however, given the single-centre design, modest sample size, borderline strength of the main LEE effect, small incremental discrimination, and absence of external validation, it should currently be regarded as an exploratory, hypothesis-generating supportive tool rather than an instrument for widespread clinical application. Larger multicentre studies are required to externally validate its discrimination, calibration, and clinical utility across different ICU populations before routine use can be recommended. Early LEE assessment may help identify patients at increased risk and support closer monitoring of multi-organ dysfunction, but whether LEE-informed clinical strategies can modify clinical decisions or improve patient-centred outcomes requires evaluation in prospective interventional studies.

## Figures and Tables

**Table 1:** Baseline demographic, clinical, and laboratory characteristics of the study population (N = 274). Continuous variables are reported as median and interquartile range (IQR), while categorical variables are expressed as absolute numbers and percentages. The table summarizes comorbidities, admission diagnoses, liver enzyme levels, LEE grade at admission and at 72 h, ALBI grade distribution, and other relevant biochemical parameters. Chronic liver disease refers to documented non-end-stage chronic liver disease; patients with end-stage chronic liver disease were excluded according to the predefined exclusion criteria.

	Total, N = 274; Median	(%) [IQR]
Age (Years)	60	[47–73]
**Gender**		
Male	183	(66.79)
Female	91	(33.21)
SAPS II	41.5	[33.25–55]
**Admission diagnosis**		
Sepsis/Abdomen	34	(12.41)
Cardiovascular	42	(15.33)
Respiratory	75	(27.37)
Neurologic	49	(17.88)
Trauma	65	(23.72)
Scheduled surgery	4	(1.46)
Other	5	(1.82)
BMI (median [IQR])	27.3	[24.5–30.9]
Charlson Comorbidity Index (median [IQR])	2	[1–4]
**Comorbidities**		
Hypertension	116	(42.34)
Asthma	10	(3.65)
COPD	65	(23.72)
Diabetes	46	(16.79)
Chronic Heart Failure	66	(24.09)
Chronic Kidney Disease	24	(8.76)
Chronic Liver Disease	13	(4.74)
Malignancy	31	(11.31)
**Obesity**		
No	196	(71.5%)
Grade 1	44	(16.1%)
Grade 2	12	(4.4%)
Grade 3	19	(6.9%)
Not reported	3	(1.1%)
Baseline AST (median [IQR])	41	[22–79]
Baseline ALT (median [IQR])	27.5	[15.3–54.0]
AST/ALT ratio (AAR) (median [IQR])	1.5	[1.1–2.1]
**LEE grade at admission (t0)**		
0	151	55.1%
1	61	22.3%
2	31	11.3%
3	13	4.7%
4	18	6.6%
AST max 72 h (median [IQR])	50	[27–113.2]
ALT max 72 h (median [IQR])	31	[17–64.5]
**LEE grade max**		
0	125	45.6%
1	63	23.0%
2	40	14.6%
3	21	7.7%
4		9.1%
HH	16	5.8%
Total bilirubin (mg/dL)	0.7	[0.4–1.1]
Direct bilirubin (mg/dL)	0.4	[0.3–0.7]
GGT (U/L)	28	[15–55.8]
Albumin (g/dL)	3.2	[2.7–3.6]
**ALBI score**		
Grade 1	43	(15.69)
Grade 2	174	(63.5)
Grade 3	56	(20.44)
Creatinine (mg/dL)	1	[0.8–1.7]
Sodium (mmol/L)	141	[138–144]
Total proteins (g/dL)	5.5	[4.8–6]
INR	1.1	[1–1.3]
Platelets (×10^3^/µL)	173	[128–236]

**Table 2:** Operational definition of Liver Enzyme Elevation (LEE) grading used in the study. LEE grade was assigned according to the highest grade reached by either AST or ALT relative to the local upper limit of normal (ULN). The local laboratory ULN was 37 U/L for AST and 55 U/L for ALT. When AST and ALT corresponded to different grades, the highest grade was retained as the final LEE grade. Bilirubin, albumin, γ-glutamyltransferase, platelet count, INR, AST/ALT ratio, and ALBI score were not used to assign LEE grade and were analysed separately as liver-related variables.

LEE Grade	Relative Ast/ALT Threshold	AST Threshold, U/L	ALT Threshold, U/L
0	Both AST and Alt <1.25 × ULN	<46.25	<68.75
1	1.25–2.5 × ULN	46.25–92.5	68.75–137.5
2	>2.5–5 × ULN	>92.5–185	>137.5–275
3	>5–10 × ULN	>185–370	>275–550
4	>10 × ULN	>370	>550

**Table 3:** Summary of major clinical outcomes in the study cohort, including mortality, ICU length of stay, use and duration of invasive mechanical ventilation, and need for renal replacement therapy.

Outcome	Value (%) [IQR]
ICU mortality	76 (27.7%)
ICU length of stay (days)	14.5 [7–26]
Duration of mechanical ventilation (days, ventilated only)	10.0 [4–20]
Dialysis (RRT)	43 (15.7%)

**Table 4:** Comparison of demographic variables, severity scores, admission diagnoses, comorbidities, and biochemical markers between survivors (n = 198) and non-survivors (n = 76). Continuous variables are reported as median and interquartile range (IQR), while categorical variables are expressed as counts and percentages. Significant differences were observed for age, SAPS II, Charlson Comorbidity Index, several comorbidities (hypertension, COPD, chronic heart failure), baseline liver enzymes, LEE grade at admission and at 72 h, presence of hypoxic hepatitis, and selected laboratory parameters. *p* values refer to comparisons between the two groups.

Variable	Survivors (n = 198; Median % [IQR])	Non-Survivors (n = 76; Median % [IQR])	*p*-Value
Age (Years)	57 [42–69]	68.5 [54–76]	<0.001
**Gender**			0.878
Male	134 (67.7%)	50 (65.8%)	
Female	64 (32.3%)	26 (34.2%)	
SAPS II	38.5 [30–49.8]	50.5 [40.8–62.5]	<0.001
**Admission diagnosis**			0.039
Sepsis/Abdomen	21 (10.6%)	13 (17.1%)	
Cardiovascular	25 (12.6%)	17 (22.4%)	
Respiratory	54 (27.3%)	21 (27.6%)	
Neurologic	35 (17.7%)	14 (18.4%)	
Trauma	55 (27.8%)	10 (13.2%)	
Scheduled surgery	4 (2.0%)	0 (0.0%)	
Other	4 (2.0%)	1 (1.3%)	
BMI	27.3 [24.2–30.2]	27.4 [24.6–32.2]	0.303
Charlson Comorbidity Index	2.0 [0.0–4.0]	4.0 [2.0–5.0]	<0.001
**Comorbidities**			
Hypertension	72 (36.4%)	44 (57.9%)	0.002
Asthma	8 (4.0%)	2 (2.6%)	0.731
COPD	39 (19.7%)	26 (34.2%)	0.018
Diabetes	31 (15.7%)	15 (19.7%)	0.530
Chronic Heart Failure	37 (18.7%)	29 (38.2%)	0.001
Chronic Kidney Disease	14 (7.1%)	10 (13.2%)	0.175
Chronic Liver Disease	9 (4.5%)	4 (5.3%)	0.759
Malignancy	21 (10.6%)	10 (13.2%)	0.701
**Obesity**			0.252
No	145 (74.4%)	51 (67.1%)	
Grade 1	32 (16.4%)	12 (15.8%)	
Grade 2	8 (4.1%)	4 (5.3%)	
Grade 3	10 (5.1%)	9 (11.8%)	
Not reported	3	0	
Baseline AST	36.5 [22–69.8]	58.5 [25.8–160]	0.009
Baseline ALT	25 [15–49.8]	38.5 [17–92.8]	0.007
AST/ALT ratio	1.5 [1.1–2.1]	1.5 [1–2]	0.620
**LEE grade at admission (t0)**			0.012
0	117 (59.1%)	34 (44.7%)	
1	44 (22.2%)	17 (22.4%)	
2	22 (11.1%)	9 (11.8%)	
3	8 (4.0%)	5 (6.6%)	
4	7 (3.5%)	11 (14.5%)	
AST max 72 h	48 [26–96.8]	66.5 [28.8–216.2]	0.026
ALT max 72 h	28.5 [16–57.8]	39.5 [20.8–118.8]	0.009
**LEE grade max**			0.008
0	95 (48.0%)	30 (39.5%)	
1	45 (22.7%)	18 (23.7%)	
2	34 (17.2%)	6 (7.9%)	
3	12 (6.1%)	9 (11.8%)	
4	12 (6.1%)	13 (17.1%)	
HH	7 (3.5%)	9 (11.8%)	0.009
Total bilirubin (mg/dL)	0.7 [0.4–1.1]	0.7 [0.5–1.3]	0.306
Direct bilirubin (mg/dL)	0.4 [0.2–0.6]	0.5 [0.3–1]	0.048
GGT (U/L)	26.5 [15–54]	32 [19.2–66.5]	0.109
Albumin (g/dL)	3.1 [2.7–3.6]	3.2 [2.6–3.6]	0.734
**ALBI score**			0.194
Grade 1	28 (14.2%)	15 (19.7%)	
Grade 2	132 (67.0%)	42 (55.3%)	
Grade 3	37 (18.8%)	19 (25.0%)	
Creatinine (mg/dL)	1.1 [0.8–1.7]	0.9 [0.73–1.22]	0.007
Sodium (mmol/L)	141 [138–144]	141 [137–144.5]	0.801
INR	1.2 [1.1–1.3]	1.1 [1–1.2]	0.049
Platelets (×10^3^/uL)	172 [122–223]	190 [138.5–256.5]	0.053

**Table 5:** Multivariable logistic regression analysis for ICU mortality including SAPS II and LEE grade at ICU admission (t0). Multivariable logistic regression model evaluating the independent association between disease severity (SAPS II) and liver enzyme elevation severity at ICU admission (LEE grade t0) with ICU mortality. Results are reported as odds ratios (OR) with 95% confidence intervals (CIs). Odds ratios represent the increase in odds of ICU mortality per one-unit increase in each variable.

Variable	OR	95% CI	*p*-Value
SAPS II, per 1-point increase	1.04	1.02–1.06	<0.001
Admission LEE grade, per 1-grade increase	1.26	1.00–1.58	0.048

## Data Availability

Data will be made available at reasonable request.

## Supplementary Materials

The following supporting information can be downloaded at: https://www.mdpi.com/article/10.3390/jcm15145513/s1, Figure S1: Adjusted marginal effects of LEE grade at ICU admission (t0) on ICU mortality, stratified by SAPS II severity. The figure shows adjusted predicted probabilities of ICU mortality according to increasing LEE grade at ICU admission, stratified into low, medium, and high SAPS II categories. Estimates were derived from multivariable logistic regression models adjusted for SAPS II, and are presented with point estimates connected by lines to illustrate trends across LEE grades; Figure S2: Forest plot showing adjusted odds ratios (ORs) with 95% confidence intervals for variables included in the multivariable logistic regression model for ICU mortality. SAPS II and age were independently associated with increased mortality, whereas severe liver enzyme elevation (LEE grade 4), hypoxic-ischemic hepatitis, and sex showed non-significant associations, with confidence intervals crossing unity; Figure S3: Forest plots showing the association between Liver Enzyme Elevation (LEE) grade and ICU mortality. The left panel depicts odds ratios (ORs) for LEE grade assessed at ICU admission (t0), while the right panel shows ORs for maximum LEE grade observed during the ICU stay. LEE grade is modelled both as an ordinal variable (per one-grade increase) and as a categorical variable, with grade 0 as the reference. Points represent adjusted ORs and horizontal bars indicate 95% confidence intervals. The vertical dashed line denotes an OR of 1. Models are adjusted for SAPS II; categorical models additionally include the same covariates as specified in the Methods. Admission LEE grade shows a more consistent graded association with ICU mortality compared with peak LEE grade; Figure S4: Decision curve analysis comparing the clinical net benefit of the SAPS II–only model and the combined SAPS II plus LEE grade at ICU admission model for prediction of ICU mortality. Across a wide range of threshold probabilities (approximately 0.15–0.40), the combined model consistently yielded a higher net benefit than SAPS II alone and outperformed both treat-all and treat-none strategies, indicating improved clinical usefulness for risk stratification. The lower axis shows the corresponding cost–benefit ratios; Figure S5: Proportion of patients requiring renal replacement therapy (RRT) across incremental LEE grades at admission (t0) and at peak severity within 72 hours (LEE max). Both trends show a progressive rise in the frequency of RRT with increasing liver injury severity. For LEE t0, RRT occurred in 8.1% of grade 0, 14.0% of grade 1, 7.1% of grade 2, 25.0% of grade 3, and 37.5% of grade 4 patients. Similarly, for LEE max the proportion requiring RRT rose from 6.3% (grade 0) to 15.3% (grade 1), 7.9% (grade 2), 22.2% (grade 3), and 31.8% (grade 4). Lines represent point estimates, with 95% confidence intervals displayed as error bars; Figure S6: Boxplots illustrating ICU length of stay across LEE grades at admission (t0). Individual patient values are shown as jittered points, and a smoothed trend line is overlaid to depict the overall pattern. Although variability within each group was high, a modest tendency toward longer ICU stay was observed with increasing LEE grade, particularly among patients with grade 4; Figure S7: Boxplots showing ICU length of stay stratified by the maximum LEE grade reached during the first 72 hours. Individual patient observations are displayed as jittered points, and a smoothed trend line is superimposed to illustrate the overall pattern. Although variability was substantial across all categories, patients with higher peak LEE grades tended to exhibit slightly longer ICU stays; Figure S8: Comparative marginal effects of admission versus peak LEE grade on ICU length of stay. Combined marginal effects plot showing predicted ICU LOS across LEE grades at admission (t0) and peak LEE grades within 72 hours, adjusted for age. Admission LEE exhibited a steeper and more consistent gradient in predicted LOS, whereas the effect of peak LEE was attenuated, supporting admission LEE as the more robust predictor of ICU stay duration. Table S1: Extent of missing data for variables included in the analyses. For each variable, the total number of patients, number of available observations, number and percentage of missing observations. Missingness was generally low. Variables included in the primary prognostic model and key variables required for LEE grading were complete in the final analytical cohort; Table S2: Results of univariate logistic regression assessing the association between clinical, biochemical, and liver-related variables and ICU mortality. Odds ratios (ORs) with 95% confidence intervals (CI) and p-values are reported. Several predictors, including SAPS II, age, Charlson Comorbidity Index, chronic heart failure, hypertension, severe liver injury (LEE grade 4), hypoxic–ischemic hepatitis, COPD, and peak liver enzymes, showed significant associations with mortality; Table S3: Results of the multivariable logistic regression model evaluating independent predictors of ICU mortality. Variables included in the model were selected a priori based on clinical relevance and univariate significance. Adjusted odds ratios (ORs), 95% confidence intervals (CI), and p-values are reported. Age and SAPS II remained independently associated with ICU mortality, whereas severe liver injury (LEE grade 4) and hypoxic–ischemic hepatitis did not retain statistical significance after adjustment; Table S4: Multivariable logistic regression analysis for ICU mortality including SAPS II and maximum LEE grade during ICU stay. Multivariable logistic regression model assessing the independent association between disease severity (SAPS II) and the maximum liver injury severity observed during ICU stay (LEE grade max) with ICU mortality. Results are expressed as odds ratios (OR) with 95% confidence intervals (CI). Odds ratios represent the increase in odds of ICU mortality per one-unit increase in each variable; Table S5: Sensitivity analysis of the association between LEE grade at ICU admission and ICU mortality. Multivariable logistic regression model including SAPS II and LEE t0 modelled as a categorical variable (LEE 0 as reference). Odds ratios are reported with 95% confidence intervals; Table S6: Diagnostic performance of the SAPS II plus LEE grade t0 model for ICU mortality prediction at the optimal cutoff identified by the Youden index. Sensitivity, specificity, positive predictive value (PPV), and negative predictive value (NPV) are reported together with the confusion matrix components; Table S7: Quantitative calibration and performance metrics of the primary SAPS II plus admission LEE grade model for ICU mortality prediction. Apparent and bootstrap optimism-corrected estimates are reported for discrimination, calibration, and overall prediction error, including AUC, calibration intercept, calibration slope, and Brier score. Bootstrap-corrected estimates were obtained using 1000 resamples; Table S8: Composition of the expanded admission-based sensitivity cohort. The table compares the original 72-hour cohort, patients excluded because of ICU length of stay shorter than 72 hours, and the expanded admission-based cohort including all otherwise eligible ICU admissions irrespective of ICU length of stay. ICU mortality and survival are reported as absolute numbers and percentages; Table S9: Sensitivity analysis of the association between admission LEE grade and ICU mortality in the expanded admission-based cohort. Multivariable logistic regression was performed in all otherwise eligible ICU admissions irrespective of ICU length of stay. The model included SAPS II and admission Liver Enzyme Elevation (LEE) grade. Results are reported as odds ratios (ORs), 95% confidence intervals (CI), and p-values. Admission LEE grade was calculated using admission AST and ALT values according to predefined upper-limit-of-normal thresholds; Table S10: Discriminative performance of SAPS II alone and SAPS II plus admission LEE grade in the expanded admission-based sensitivity cohort. Receiver operating characteristic analysis was performed in all otherwise eligible ICU admissions irrespective of ICU length of stay. AUC values are reported with 95% confidence intervals. The incremental value of admission LEE grade was assessed by comparing correlated ROC curves using DeLong’s test; Table S11: Sensitivity analyses accounting for trauma status and admission diagnosis heterogeneity. Logistic regression models were used to evaluate whether the association between admission Liver Enzyme Elevation (LEE) grade and ICU mortality was influenced by the trauma subgroup. Trauma status was modelled as a binary variable because several admission diagnosis categories were small. Models included the primary 72-hour cohort, a trauma-adjusted model, a non-trauma-only analysis, an exploratory interaction model, and an expanded admission-based sensitivity model including all otherwise eligible ICU admissions irrespective of ICU length of stay; Table S12: Univariate logistic regression for predictors of renal replacement therapy (RRT) after exclusion of patients with chronic renal failure at baseline. Odds ratios (OR) with 95% confidence intervals (CI) are reported. Markers of hepatic injury (LEE grade at admission, peak LEE grade, hypoxic–ischemic hepatitis, and peak transaminases), overall illness severity (SAPS II), and metabolic factors (obesity, BMI) were significantly associated with the need for dialysis; Table S13: Logistic regression models evaluating baseline and peak LEE grade as predictors of RRT (N=249); Table S14: Multivariable logistic regression models for predictors of dialysis (RRT). Four multivariable models were constructed to evaluate the independent association between hepatic injury (LEE grade) and the need for dialysis. Models A1 and A2 included BMI as a continuous covariate, whereas Models B1 and B2 used obesity as a categorical predictor. LEE severity was entered either as grade at admission (t0) or as peak grade within 72 hours (max). All models were adjusted for SAPS II and diabetes. Results are presented as adjusted odds ratios (ORs) with 95% confidence intervals; Table S15: Sensitivity Analysis: logistic regression models evaluating the association between LEE severity and the need for dialysis, adjusted only for SAPS II. Both LEE grade at admission and peak LEE grade remained significantly associated with dialysis, confirming the robustness of the effect after minimal adjustment for overall illness severity; Table S16: Univariate log-linear regression evaluating the association between clinical and hepatic injury variables and ICU length of stay (LOS) in the overall cohort. Regression coefficients (β), 95% confidence intervals (CI), p-values, and the corresponding percentage change in LOS are reported. Positive β values indicate longer LOS, whereas negative coefficients indicate shorter LOS; Table S17: Multivariable regression assessing the independent association between LEE grade at admission (t0) and ICU length of stay (LOS), adjusted for SAPS II. Regression coefficients (β), 95% confidence intervals (CI), p-values, and corresponding percent change in LOS are reported. Positive β values indicate longer ICU stay; Table S18: Multivariable regression evaluating the association between maximum LEE grade reached in the first 72 hours and ICU length of stay (LOS), adjusted for SAPS II. Reported values include regression coefficients (β), 95% confidence intervals (CI), p-values, and corresponding percent change in LOS. Positive β values indicate longer ICU stay; Table S19: Univariate log-linear regression analyses evaluating the association between clinical characteristics, comorbidities, severity of illness, and hepatic injury markers with ICU length of stay (LOS) among survivors. Positive β coefficients indicate longer LOS, whereas negative coefficients reflect shorter LOS. Both LEE grade at admission and peak LEE grade within 72 hours were significantly associated with prolonged ICU stay; Table S20: Multivariable analysis restricted to survivors, evaluating the independent association between LEE grade at admission (t0) and ICU length of stay (LOS), adjusted for age. Regression coefficients (β), approximate 95% confidence intervals (CI), p-values, and model interpretations are reported. Positive β values indicate longer ICU stay; Table S21: Multivariable analysis restricted to survivors, assessing whether the maximum LEE grade reached within the first 72 hours (peak LEE max) is independently associated with ICU length of stay (LOS) after adjustment for age. Regression coefficients (β), approximate 95% confidence intervals (CI), p-values, and clinical interpretations are reported. Positive β coefficients indicate longer ICU stay; Table S22: Multivariable model restricted to survivors and excluding HH, evaluating the independent association between LEE grade at admission (t0) and ICU length of stay (LOS), adjusted for age. Coefficients (β), standard errors, t values and p-values are reported. Positive β coefficients indicate longer LOS; Table S23: Multivariable analysis restricted to ICU survivors and excluding patients with hypoxic–ischemic hepatitis (HH), evaluating whether the maximum LEE grade reached within the first 72 hours (peak LEE max) is independently associated with ICU length of stay (LOS) after adjustment for age. Regression coefficients (β), approximate 95% confidence intervals (CI), p-values, and percent change in LOS are reported. Positive β coefficients indicate longer LOS; Table S24: Model evaluating the independent association of log-transformed AST and ALT at ICU admission with ICU length of stay (LOS) among survivors. Regression coefficients (β), approximate 95% confidence intervals, p-values, and percentage change in LOS (exp(β) − 1) are reported. Positive values indicate longer LOS; Table S25: Sensitivity analysis restricted to ICU survivors: multivariable log-linear regression assessing the association of peak AST and ALT within 72 hours with ICU length of stay (LOS), adjusted for age. Regression coefficients (β), 95% confidence intervals, and percent change in LOS are reported. Positive β coefficients represent longer ICU LOS. Neither AST nor ALT peak values were independently associated with LOS; Table S26: Univariate log-linear regression analyses evaluating the association between clinical variables and duration of invasive mechanical ventilation among ICU survivors. Positive β coefficients indicate longer ventilation duration; Table S27: Multivariable log-linear regression model evaluating the independent association of obesity class and LEE grade at admission with the duration of invasive mechanical ventilation among ICU survivors. Positive coefficients indicate longer ventilation time. Obesity and LEE grade at admission were both independently associated with prolonged ventilation; Table S28: Multivariable log-linear regression model assessing BMI (continuous) and LEE grade at admission as predictors of invasive mechanical ventilation duration among ICU survivors. BMI remained significantly associated with longer ventilation duration, whereas the association with LEE grade at admission did not reach statistical significance; Table S29: Multivariable log-linear regression model evaluating the association of obesity class and peak LEE grade within the first 72 hours with the duration of invasive mechanical ventilation among ICU survivors. Obesity remained a strong independent predictor, while peak LEE grade did not demonstrate a significant association; Table S30: Multivariable log-linear regression model assessing BMI (continuous) and peak LEE grade as predictors of invasive mechanical ventilation duration among ICU survivors. BMI remained significantly associated with ventilation duration, whereas peak LEE grade did not show an independent effect.
