# Supplementary material for: Admission Liver Enzyme Elevation Grade for Risk Stratification in Critically Ill Patients: Development and Internal Validation of an Exploratory Prognostic Model
Source: J Clin Med. 2026 Jul 14;15(14):5513. doi: 10.3390/jcm15145513 (PMC13413069; doi:10.3390/jcm15145513)
Supplement: Supplementary file 1 [file jcm-15-05513-s001.zip › jcm-4395501-supplementary.pdf]

Supplemental Material

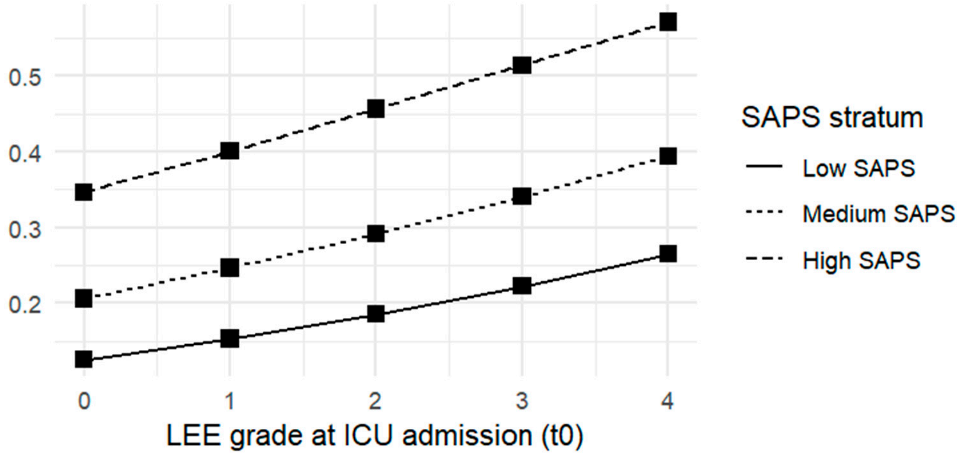

Supplementary Figure S1. Adjusted marginal effects of LEE grade at ICU admission (t0) on ICU mortality, stratified by SAPS II severity. The figure shows adjusted predicted probabilities of ICU mortality according to increasing LEE grade at ICU admission, stratified into low, medium, and high SAPS II categories. Estimates were derived from multivariable logistic regression models adjusted for SAPS II, and are presented with point estimates connected by lines to illustrate trends across LEE grades.

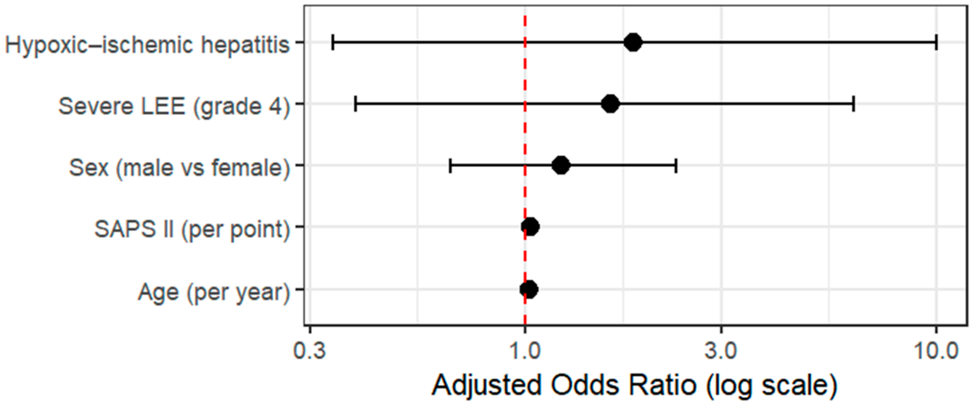

Supplementary Figure S2. Forest plot showing adjusted odds ratios (ORs) with 95% confidence intervals for variables included in the multivariable logistic regression model for ICU mortality. SAPS II and age were independently associated with increased mortality, whereas severe liver enzyme elevation (LEE grade 4), hypoxic-ischemic hepatitis, and sex showed non-significant associations, with confidence intervals crossing unity.

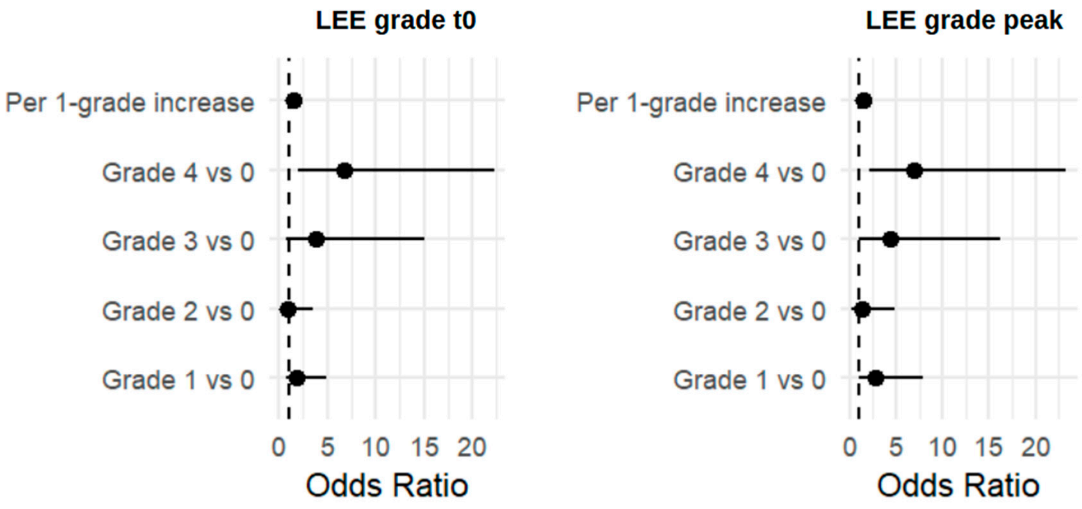

Supplementary Figure S3. Forest plots showing the association between Liver Enzyme Elevation (LEE) grade and ICU mortality. The left panel depicts odds ratios (ORs) for LEE grade assessed at ICU admission (t0), while the right panel shows ORs for maximum LEE grade observed during the ICU stay. LEE grade is modelled both as an ordinal variable (per one-grade increase) and as a categorical variable, with grade 0 as the reference. Points represent adjusted ORs and horizontal bars indicate 95% confidence intervals. The vertical dashed line denotes an OR of 1. Models are adjusted for SAPS II; categorical models additionally include the same covariates as specified in the Methods. Admission LEE grade shows a more consistent graded association with ICU mortality compared with peak LEE grade.

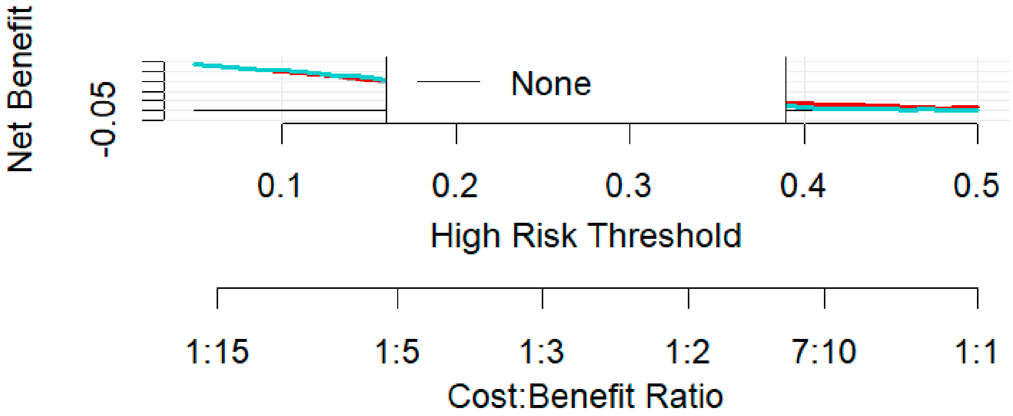

Supplementary Figure S4. Decision curve analysis comparing the clinical net benefit of the SAPS II–only model and the combined SAPS II plus LEE grade at ICU admission model for prediction of ICU mortality. Across a wide range of threshold probabilities

(approximately 0.15–0.40), the combined model consistently yielded a higher net benefit than SAPS II alone and outperformed both treat-all and treat-none strategies, indicating improved clinical usefulness for risk stratification. The lower axis shows the corresponding cost–benefit ratios.

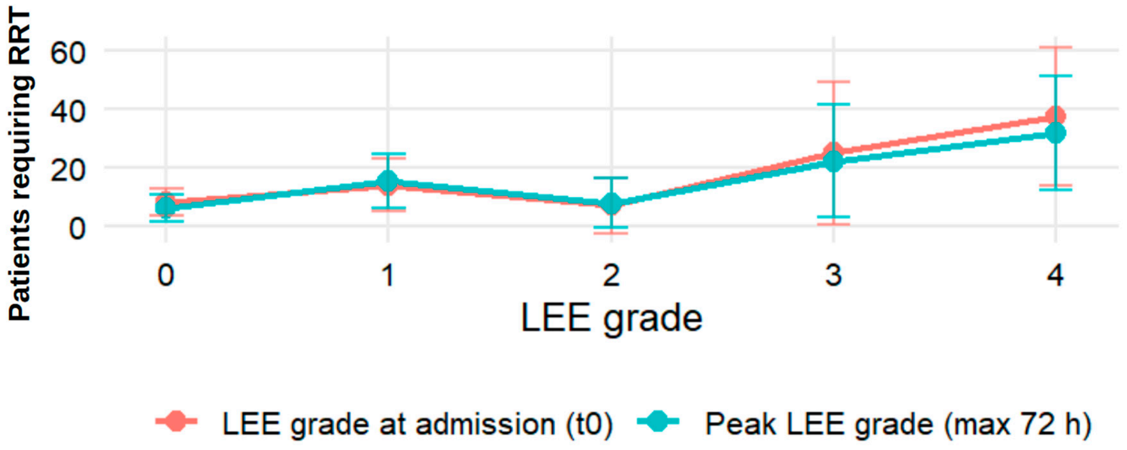

Supplementary Figure S5. Proportion of patients requiring renal replacement therapy (RRT) across incremental LEE grades at admission (t0) and at peak severity within 72 hours (LEE max). Both trends show a progressive rise in the frequency of RRT with increasing liver injury severity. For LEE t0, RRT occurred in 8.1% of grade 0, 14.0% of grade 1, 7.1% of grade 2, 25.0% of grade 3, and 37.5% of grade 4 patients. Similarly, for LEE max the proportion requiring RRT rose from 6.3% (grade 0) to 15.3% (grade 1), 7.9% (grade 2), 22.2% (grade 3), and 31.8% (grade 4). Lines represent point estimates, with 95% confidence intervals displayed as error bars.

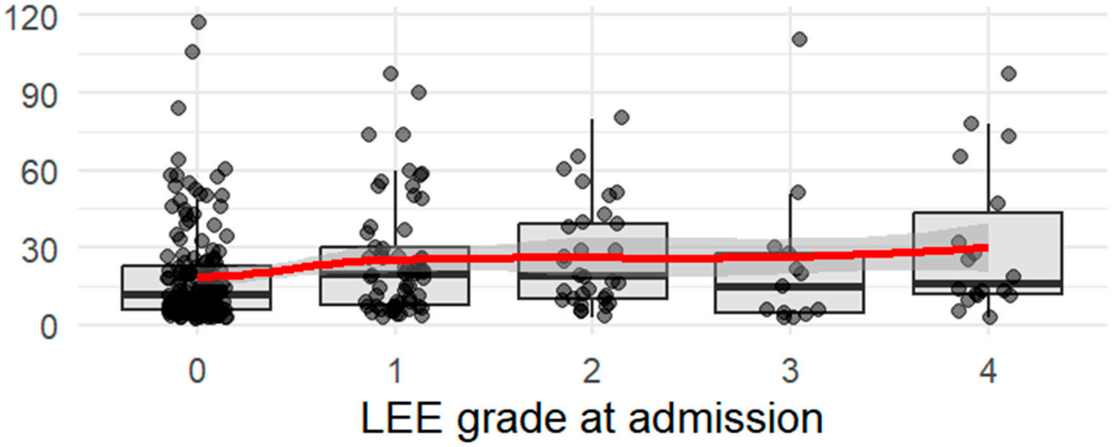

Supplementary Figure S6. Boxplots illustrating ICU length of stay across LEE grades at admission (t0). Individual patient values are shown as jittered points, and a smoothed trend line is overlaid to depict the overall pattern. Although variability within each group

was high, a modest tendency toward longer ICU stay was observed with increasing LEE grade, particularly among patients with grade 4.

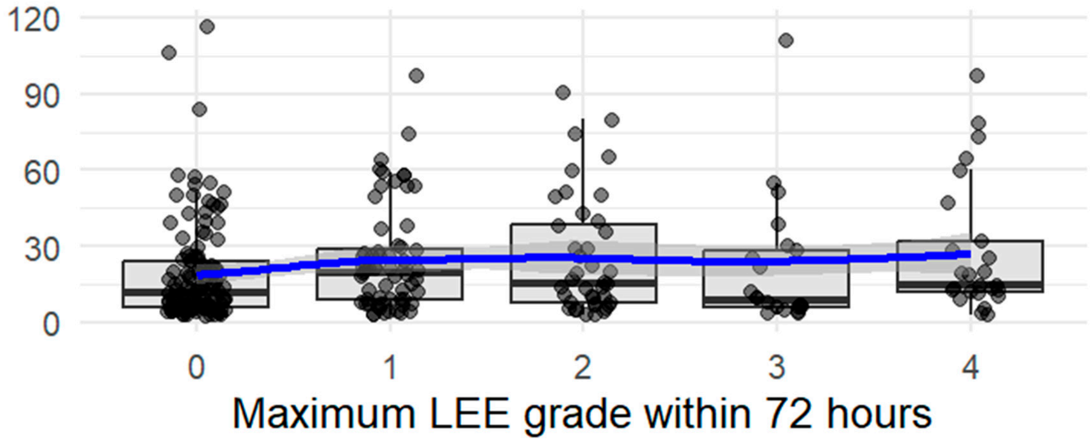

Supplementary Figure S7. Boxplots showing ICU length of stay stratified by the maximum LEE grade reached during the first 72 hours. Individual patient observations are displayed as jittered points, and a smoothed trend line is superimposed to illustrate the overall pattern. Although variability was substantial across all categories, patients with higher peak LEE grades tended to exhibit slightly longer ICU stays.

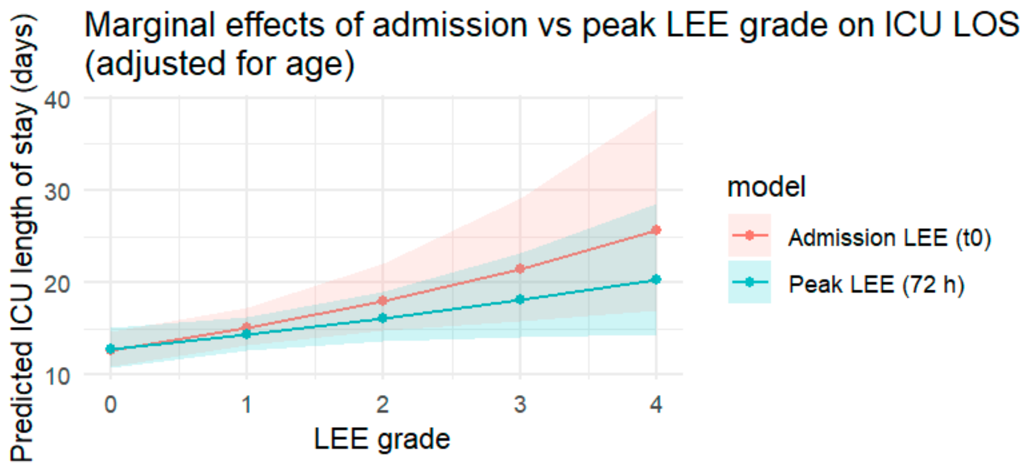

Supplementary Figure S8. Comparative marginal effects of admission versus peak LEE grade on ICU length of stay. Combined marginal effects plot showing predicted ICU LOS across LEE grades at admission (t0) and peak LEE grades within 72 hours, adjusted for age. Admission LEE exhibited a steeper and more consistent gradient in predicted LOS, whereas the effect of peak LEE was attenuated, supporting admission LEE as the more robust predictor of ICU stay duration.

Supplementary Table S1 Extent of missing data for variables included in the analyses. For each variable, the total number of patients, number of available observations, number and percentage of missing observations. Missingness was generally low. Variables included in the primary prognostic model and key variables required for LEE grading were complete in the final analytical cohort

Variable	N total	N available	Missing, n	Missing, %
Study ID	274	274	0	0,0
Date of birth	274	274	0	0,0
Age	274	274	0	0,0
Sex	274	274	0	0,0
SAPS II	274	274	0	0,0
Hospital admission date	274	274	0	0,0
ICU admission date	274	274	0	0,0
ICU discharge date	274	274	0	0,0
Hospital discharge date	274	273	1	0,4
Primary diagnosis	274	274	0	0,0
Admission diagnosis category I	274	274	0	0,0
Admission diagnosis category II	274	274	0	0,0
AIS liver score (trauma patients)	274	274	0	0,0
Weight	274	271	3	1,1
Height	274	271	3	1,1
BMI	274	274	0	0,0
Obesity class	274	271	3	1,1
Hypertension	274	273	1	0,4
Asthma	274	273	1	0,4
COPD	274	273	1	0,4
Diabetes	274	273	1	0,4
Pregnancy	274	273	1	0,4
Chronic heart failure	274	273	1	0,4
Chronic kidney disease	274	273	1	0,4
Chronic liver disease	274	273	1	0,4
Oral anticoagulant therapy	274	273	1	0,4
Malignancy	274	273	1	0,4
Autoimmune disorder	274	273	1	0,4
Immunosuppression	274	273	1	0,4
Peripheral vascular disease	274	273	1	0,4
Alcohol use disorder	274	273	1	0,4
Substance use disorder	274	273	1	0,4
Charlson Comorbidity Index	274	273	1	0,4
Baseline AST	274	274	0	0,0
Baseline ALT	274	274	0	0,0

AST/ALT ratio	274	274	0	0,0
LEE grade at admission	274	274	0	0,0
Peak AST within 72 h	274	274	0	0,0
Peak ALT within 72 h	274	274	0	0,0
Peak LEE grade within 72 h	274	274	0	0,0
Hypoxic-ischemic hepatitis within 72 h	274	274	0	0,0
Total bilirubin	274	273	1	0,4
Total bilirubin (μmol/L)	274	274	0	0,0
Direct bilirubin	274	266	8	2,9
GGT	274	270	4	1,5
Albumin	274	268	6	2,2
Albumin (g/L)	274	274	0	0,0
ALBI score	274	274	0	0,0
ALBI grade	274	274	0	0,0
Creatinine	274	274	0	0,0
Sodium	274	273	1	0,4
Total proteins	274	257	17	6,2
INR	274	268	6	2,2
Platelets	274	273	1	0,4
ICU length of stay	274	274	0	0,0
Hospital length of stay	274	273	1	0,4
Duration of invasive mechanical ventilation	274	272	2	0,7
Discharged while mechanically ventilated	274	274	0	0,0
ICU mortality	274	274	0	0,0
Hospital mortality	274	274	0	0,0
Septic shock	274	274	0	0,0
Noradrenaline requirement	274	273	1	0,4
Inotrope requirement	274	273	1	0,4
Renal replacement therapy	274	274	0	0,0
VV-ECMO	274	274	0	0,0
VA-ECMO	274	274	0	0,0

Supplementary Table S2. Results of univariate logistic regression assessing the association between clinical, biochemical, and liver-related variables and ICU mortality. Odds ratios (ORs) with 95% confidence intervals (CI) and p-values are reported. Several predictors, including SAPS II, age, Charlson Comorbidity Index, chronic heart failure, hypertension,

severe liver injury (LEE grade 4), hypoxic–ischemic hepatitis, COPD, and peak liver enzymes, showed significant associations with mortality.

Predictor	OR	95% CI	p-value
SAPS II (per point)	**1.05**	1.03–1.06	**2.0×10^{-7}**
Age (per year)	**1.04**	1.02–1.05	**7.7×10^{-5}**
Charlson index (per point)	**1.28**	1.13–1.45	**1.7×10^{-4}**
Chronic heart failure	**2.67**	1.48–4.79	**0.0010**
Hypertension	**2.39**	1.40–4.12	**0.0016**
Severe liver injury (LEE grade 4)	**3.20**	1.38–7.47	**0.0063**
Hypoxic–ischemic hepatitis	**3.67**	1.32–10.62	**0.013**
COPD	**2.11**	1.16–3.79	**0.013**
Peak ALT (72h)	**1.00**	1.00–1.00	**0.016**
Peak AST (72h)	**1.00**	1.00–1.00	**0.018**
Platelets ($\times 10^3/\mu L$)	**1.00**	1.00–1.01	**0.029**
Baseline ALT	**1.00**	1.00–1.00	**0.042**
Baseline AST	1.00	1.00–1.00	0.072
Creatinine	0.77	0.56–1.01	0.075
Direct bilirubin	1.19	0.99–1.52	0.113
INR	0.55	0.20–0.94	0.179
Total bilirubin	1.11	0.94–1.35	0.224
ALBI score	0.92	0.73–1.05	0.297
Albumin	1.09	0.97–1.48	0.305
GGT	1.00	1.00–1.00	0.567
Total proteins	0.98	0.80–1.07	0.691
Sex (male vs female)	0.92	0.53–1.62	0.766
Baseline AST/ALT ratio	0.97	0.70–1.31	0.845
Sodium	1.00	0.95–1.05	0.962

Supplementary Table S3. Results of the multivariable logistic regression model evaluating independent predictors of ICU mortality. Variables included in the model were selected a priori based on clinical relevance and univariate significance. Adjusted odds ratios (ORs), 95% confidence intervals (CI), and p-values are reported. Age and SAPS II remained independently associated with ICU mortality, whereas severe liver injury (LEE grade 4) and hypoxic–ischemic hepatitis did not retain statistical significance after adjustment.

Predictor	Adjusted OR	95% CI	p-value
Age (per year)	1.025	1.01 – 1.05	0.018
Sex (male vs female)	1.23	0.66 – 2.33	0.531
SAPS II (per point)	1.036	1.02 – 1.06	0.00025
Severe liver injury (LEE grade 4)	1.62	0.39 – 6.31	0.490
Hypoxic–ischemic hepatitis (HH)	1.84	0.34 – 10.03	0.481

Supplementary Table S4. Multivariable logistic regression analysis for ICU mortality including SAPS II and maximum LEE grade during ICU stay. Multivariable logistic regression model assessing the independent association between disease severity (SAPS II) and the maximum liver injury severity observed during ICU stay (LEE grade max) with ICU mortality. Results are expressed as odds ratios (OR) with 95% confidence intervals (CI). Odds ratios represent the increase in odds of ICU mortality per one-unit increase in each variable.

Variabile	OR	IC 95%	p-value
SAPS II (per punto)	**1.04**	1.03 – 1.06	<0.001
LEE grade max (per classe)	1.17	0.95 – 1.44	0.143

Supplementary Table S5. Sensitivity analysis of the association between LEE grade at ICU admission and ICU mortality. Multivariable logistic regression model including SAPS II and LEE t0 modelled as a categorical variable (LEE 0 as reference). Odds ratios are reported with 95% confidence intervals.

**Variabile**	**OR**	**IC 95%**	**p**
SAPS	1.04	1.02–1.06	<0.001
LEE t0 = 1 vs 0	1.22	0.58–2.50	0.586
LEE t0 = 2 vs 0	1.23	0.48–2.96	0.646
LEE t0 = 3 vs 0	2.14	0.58–7.22	0.228
LEE t0 = 4 vs 0	2.79	0.93–8.68	0.069

Supplementary Table S6. Diagnostic performance of the SAPS II plus LEE grade t0 model for ICU mortality prediction at the optimal cutoff identified by the Youden index. Sensitivity, specificity, positive predictive value (PPV), and negative predictive value (NPV) are reported together with the confusion matrix components.

Threshold	Sensitivity	Specificity	PPV	NPV	TP	FP	TN	FN
0.229	0.79	0.62	0.44	0.88	60	75	123	16

Supplementary Table S7. Quantitative calibration and performance metrics of the primary SAPS II plus admission LEE grade model for ICU mortality prediction. Apparent and bootstrap optimism-corrected estimates are reported for discrimination, calibration, and overall prediction error, including AUC, calibration intercept, calibration slope, and Brier score. Bootstrap-corrected estimates were obtained using 1000 resamples

Parameter / metric	Estimate
Intercept	−3.067
SAPS II coefficient	0.0409
Admission LEE grade coefficient	0.2299
SAPS II OR	1.04
Admission LEE grade OR	1.26
Apparent AUC	0.737
Apparent AUC 95% CI	0.674–0.799
Bootstrap optimism-corrected AUC	0.730
Apparent calibration intercept	0.000
Bootstrap optimism-corrected calibration intercept	−0.010
Apparent calibration slope	1.000
Bootstrap optimism-corrected calibration slope	0.975
Apparent Brier score	0.176
Bootstrap optimism-corrected Brier score	0.181

Supplementary Table S8. Composition of the expanded admission-based sensitivity cohort. The table compares the original 72-hour cohort, patients excluded because of ICU length of stay shorter than 72 hours, and the expanded admission-based cohort including all otherwise eligible ICU admissions irrespective of ICU length of stay. ICU mortality and survival are reported as absolute numbers and percentages.

Cohort	n	ICU deaths, n (%)	ICU survivors, n (%)
Original 72-hour cohort	274	76 (27.7%)	198 (72.3%)
Patients with ICU LOS <72 h	21	12 (57.1%)	9 (42.9%)
Expanded admission-based cohort	295	88 (29.8%)	207 (70.2%)

Supplementary Table S9. Sensitivity analysis of the association between admission LEE grade and ICU mortality in the expanded admission-based cohort. Multivariable logistic regression was performed in all otherwise eligible ICU admissions irrespective of ICU length of stay. The model included SAPS II and admission Liver Enzyme Elevation (LEE) grade. Results are reported as odds ratios (ORs), 95% confidence intervals (CI), and p-values. Admission LEE grade was calculated using admission AST and ALT values according to predefined upper-limit-of-normal thresholds.

Predictor	OR	95% CI	p-value
SAPS II, per point	1.047	1.030–1.065	<0.001
Admission LEE grade, per grade	1.306	1.046–1.629	0.018

Supplementary Table S10. Discriminative performance of SAPS II alone and SAPS II plus admission LEE grade in the expanded admission-based sensitivity cohort. Receiver operating characteristic analysis was performed in all otherwise eligible ICU admissions irrespective of ICU length of stay. AUC values are reported with 95% confidence intervals. The incremental value of admission LEE grade was assessed by comparing correlated ROC curves using DeLong's test.

Model	AUC	95% CI
SAPS II alone	0.740	0.679–0.802
SAPS II + admission LEE grade	0.770	0.714–0.826

Supplementary Table S11. Sensitivity analyses accounting for trauma status and admission diagnosis heterogeneity. Logistic regression models were used to evaluate whether the association between admission Liver Enzyme Elevation (LEE) grade and ICU mortality was influenced by the trauma subgroup. Trauma status was modelled as a binary variable because several admission diagnosis categories were small. Models included the primary 72-hour cohort, a trauma-adjusted model, a non-trauma-only analysis, an exploratory interaction model, and an expanded admission-based sensitivity model including all otherwise eligible ICU admissions irrespective of ICU length of stay.

Model	Cohort	n / deaths	Admission LEE OR	95% CI	p	AUC	Additional finding
Primary model	72h cohort	274 / 76	1.262	1.004–1.585	0.046	0.738	SAPS II + LEE
Trauma-adjusted model	72h cohort	274 / 76	1.280	1.017–1.611	0.035	0.751	Trauma OR 0.501, 95% CI 0.233–1.080, p = 0.078
Non-trauma only	72h cohort excluding trauma	209 / 66	1.181	0.920–1.515	0.192	0.753	Association attenuated
LEE × trauma interaction	72h cohort	274 / 76	Interaction OR 1.672	0.816–3.427	0.161	0.757	No clear evidence of interaction,

Model	Cohort	n / deaths	Admission LEE OR	95% CI	p	AUC	Additional finding
							underpowered
Trauma-adjusted model	Expanded cohort, all LOS	295 / 88	1.329	1.063–1.661	0.013	0.783	Trauma OR 0.487, 95% CI 0.230–1.030, p = 0.060

Supplementary Table S12 Univariate logistic regression for predictors of renal replacement therapy (RRT) after exclusion of patients with chronic renal failure at baseline. Odds ratios (OR) with 95% confidence intervals (CI) are reported. Markers of hepatic injury (LEE grade at admission, peak LEE grade, hypoxic–ischemic hepatitis, and peak transaminases), overall illness severity (SAPS II), and metabolic factors (obesity, BMI) were significantly associated with the need for dialysis.

Predictor	OR	95% CI	p-value
**Peak LEE grade (max 72h)**	**1.53**	1.17 – 2.01	**0.0017**
**LEE grade at admission (t0)**	**1.53**	1.16 – 2.02	**0.0024**
**SAPS II (per point)**	**1.03**	1.01 – 1.05	**0.0043**
**Obesity (categorical)**	**1.61**	1.10 – 2.30	**0.0114**
**Platelets (×10³/µL)**	1.00	1.00 – 1.01	**0.0141**
**Hypoxic–ischemic hepatitis (HH)**	**4.18**	1.22 – 12.8	**0.0149**
Peak ALT	1.00	1.00 – 1.00	0.0220
Peak AST	1.00	1.00 – 1.00	0.0279
**BMI**	**1.05**	1.00 – 1.10	**0.0417**
Diabetes	2.48	0.96 – 5.94	0.0482
Baseline ALT	1.00	1.00 – 1.00	0.0628

Predictor	OR	95% CI	p-value
Hypertension	1.95	0.90 – 4.25	0.089
Baseline AST	1.00	1.00 – 1.00	0.159
COPD	0.48	0.14 – 1.31	0.192
Sex (male)	1.75	0.75 – 4.57	0.220
ALBI score	1.35	0.93 – 2.19	0.226
Chronic heart failure	1.61	0.66 – 3.66	0.269
Age	1.01	0.99 – 1.04	0.330
AAR	1.21	0.77 – 1.83	0.371
Creatinine	1.12	0.79 – 1.50	0.480
Sodium	1.02	0.95 – 1.09	0.576
Chronic liver disease	1.49	0.22 – 6.04	0.617
Albumin	0.94	0.54 – 1.11	0.735
INR	0.91	0.32 – 1.02	0.814
GGT	1.00	0.995 – 1.00	0.827
Total bilirubin	0.98	0.64 – 1.19	0.855
Malignancy	0.90	0.20 – 2.81	0.874
Charlson index	1.01	0.83 – 1.22	0.931
Asthma	1.04	0.05 – 6.17	0.968
Direct bilirubin	1.00	0.68 – 1.22	0.996

Supplementary Table S13. Logistic regression models evaluating baseline and peak LEE grade as predictors of RRT (N=249).

Model	Predictor	OR	95% CI	p-value

Model	Predictor	OR	95% CI	p-value
**LEE t0 – Linear**	per 1-grade increase	**1.53**	1.16–2.02	**0.0024**
**LEE t0 – Cat.**	Grade 1 vs 0	1.86	0.71–4.03	0.211
	Grade 2 vs 0	0.87	0.16–3.53	0.866
	Grade 3 vs 0	3.79	0.94–11.4	0.071
	Grade 4 vs 0	**6.82**	2.00–18.0	**0.0015**
**Linearity test (t0)**	—	—	—	**p = 0.422**
**LEE max – Linear**	per 1-grade increase	**1.53**	1.17–2.01	**0.0018**
**LEE max – Cat.**	Grade 1 vs 0	2.70	0.95–6.98	0.062
	Grade 2 vs 0	1.29	0.29–4.47	0.726
	Grade 3 vs 0	**4.29**	1.13–12.8	**0.035**
	Grade 4 vs 0	**7.00**	2.08–17.3	**0.0012**
**Linearity test (max)**	—	—	—	**p = 0.346**

Supplementary Table S14 Multivariable logistic regression models for predictors of dialysis (RRT).

Four multivariable models were constructed to evaluate the independent association between hepatic injury (LEE grade) and the need for dialysis. Models A1 and A2 included BMI as a continuous covariate, whereas Models B1 and B2 used obesity as a categorical predictor. LEE severity was entered either as grade at admission (t0) or as peak grade within 72 hours (max). All models were adjusted for SAPS II and diabetes. Results are presented as adjusted odds ratios (ORs) with 95% confidence intervals.

Model	Predictor	Adjusted OR	95% CI	p-value

Model	Predictor	Adjusted OR	95% CI	p-value
**A1 – LEE t0 + SAPS II + BMI + diabetes**	LEE grade t0	**1.34**	1.00–1.80	**0.048**
	SAPS II (per point)	1.02	1.00–1.04	0.051
	BMI (continuous)	1.04	0.99–1.10	0.131
	Diabetes	1.99	0.73–4.99	0.157
**A2 – LEE max + SAPS II + BMI + diabetes**	LEE grade max	**1.41**	1.06–1.86	**0.017**
	SAPS II (per point)	1.02	1.00–1.04	0.053
	BMI (continuous)	1.04	0.99–1.10	0.119
	Diabetes	2.13	0.78–5.42	0.121
**B1 – LEE t0 + SAPS II + obesity + diabetes**	LEE grade t0	**1.36**	1.01–1.81	**0.040**
	SAPS II (per point)	1.02	1.00–1.04	0.055
	Obesity (categorical)	**1.50**	1.00–2.21	**0.044**
	Diabetes	1.88	0.69–4.76	0.194
**B2 – LEE max + SAPS II + obesity + diabetes**	LEE grade max	**1.43**	1.08–1.90	**0.013**

Model	Predictor	Adjusted OR	95% CI	p-value
	SAPS II (per point)	1.02	1.00–1.04	0.058
	Obesity (categorical)	**1.53**	1.01–2.27	**0.035**
	Diabetes	2.02	0.74–5.15	0.153

Supplementary Table S15. Sensitivity Analysis: logistic regression models evaluating the association between LEE severity and the need for dialysis, adjusted only for SAPS II. Both LEE grade at admission and peak LEE grade remained significantly associated with dialysis, confirming the robustness of the effect after minimal adjustment for overall illness severity.

Model	Predictor	Adjusted OR	95% CI	p-value
**LEE at admission**	**LEE grade at admission (t0)**	**1.40**	**1.04 – 1.86**	**0.022**
	SAPS II	1.02	1.00 – 1.04	0.035
**Peak LEE**	**LEE grade max (72h)**	**1.43**	**1.08 – 1.88**	**0.012**
	SAPS II	1.02	1.00 – 1.04	0.029

Supplementary Table S16. Univariate log-linear regression evaluating the association between clinical and hepatic injury variables and ICU length of stay (LOS) in the overall cohort. Regression coefficients (β), 95% confidence intervals (CI), p-values, and the

corresponding percentage change in LOS are reported. Positive β values indicate longer LOS, whereas negative coefficients indicate shorter LOS.

**Predictor**	**β coefficient**	**95% CI**	**p-value**	**% change in LOS***	**95% CI (%)**
**LEE grade at admission (t0)**	**0.122**	0.030 to 0.214	**0.0097**	**+12.9%**	+3.0% to +23.9%
**Age (per year)**	−0.00581	−0.0121 to 0.00050	0.071	−0.6%	−1.2% to +0.5%
Peak LEE grade (max 72h)	0.0752	−0.009 to 0.160	0.080	+7.8%	−0.9% to +17.4%
COPD	−0.183	−0.443 to 0.077	0.167	−16.7%	−35.8% to +8.0%
Charlson index	−0.0258	−0.0776 to 0.0261	0.328	−2.5%	−7.5% to +2.6%
Chronic heart failure	0.0700	−0.189 to 0.329	0.596	+7.3%	−17.2% to +39.0%
Hypertension	0.0220	−0.203 to 0.247	0.847	+2.2%	−18.3% to +28.1%
Creatinine	0.00161	−0.099 to 0.102	0.975	+0.16%	−9.4% to +10.7%
SAPS II	−0.000048	−0.0062 to 0.0061	0.988	−0.005%	−0.6% to +0.6%

Supplementary Table S17. Multivariable regression assessing the independent association between LEE grade at admission (t0) and ICU length of stay (LOS), adjusted for SAPS II. Regression coefficients (β), 95% confidence intervals (CI), p-values, and corresponding percent change in LOS are reported. Positive β values indicate longer ICU stay.

Variable	Beta	95% CI	p-value	% LOS change
SAPS II	-0.0019	-0.0076 to 0.0038	0.518	NS
LEE t0 (per grade)	0.120	0.033 to 0.207	0.007	+13%

Supplementary Table S18. Multivariable regression evaluating the association between maximum LEE grade reached in the first 72 hours and ICU length of stay (LOS), adjusted for SAPS II. Reported values include regression coefficients (β), 95% confidence intervals (CI), p-values, and corresponding percent change in LOS. Positive β values indicate longer ICU stay.

Variable	Beta	95% CI	p-value	% LOS change
SAPS II	-0.0010	-0.0067 to 0.0047	0.728	NS
LEE max (per grade)	0.073	-0.006 to 0.152	0.071	+7.6%

Supplementary Table S19. Univariate log-linear regression analyses evaluating the association between clinical characteristics, comorbidities, severity of illness, and hepatic injury markers with ICU length of stay (LOS) among survivors. Positive β coefficients indicate longer LOS, whereas negative coefficients reflect shorter LOS. Both LEE grade at admission and peak LEE grade within 72 hours were significantly associated with prolonged ICU stay.

Variable	β coefficient (log-LOS)	p-value
LEE grade at admission (t0)	**+0.168**	**0.0036**
Peak LEE grade (max 72h)	**+0.113**	**0.0249**
Age (per year)	–0.0066	0.0537
COPD	–0.236	0.1213
Charlson comorbidity index	–0.043	0.1477
SAPS II	+0.0053	0.1605
Creatinine	+0.033	0.5372
Chronic heart failure	+0.058	0.7085
Hypertension	–0.032	0.8015

Supplementary Table S20. Multivariable analysis restricted to survivors, evaluating the independent association between LEE grade at admission (t0) and ICU length of stay (LOS), adjusted for age. Regression coefficients (β), approximate 95% confidence intervals (CI), p-values, and model interpretations are reported. Positive β values indicate longer ICU stay.

Predictor	β	95% CI (approx.)	p-value	Interpretation
**Age (per year)**	−0.0064	−0.0139 to +0.0005	0.057	Trend toward shorter LOS with age (borderline)
**LEE grade at admission (t0)**	**+0.1654**	**+0.055 to +0.276**	**0.0038**	Each 1-point ↑ LEE t0 → **~18%** ↑ **LOS**

Supplementary Table S21. Multivariable analysis restricted to survivors, assessing whether the maximum LEE grade reached within the first 72 hours (peak LEE max) is

independently associated with ICU length of stay (LOS) after adjustment for age. Regression coefficients (β), approximate 95% confidence intervals (CI), p-values, and clinical interpretations are reported. Positive β coefficients indicate longer ICU stay.

Predictor	β	95% CI (approx.)	p-value	Interpretation
**Age (per year)**	−0.0062	−0.013 to +0.0008	0.068	Trend toward shorter LOS (NS)
**Peak LEE max (72h)**	**+0.1080**	+0.009 to +0.206	**0.0315**	Each 1-point ↑ LEE max → **~11%** ↑ LOS

Supplementary Table S22. Multivariable model restricted to survivors and excluding HH, evaluating the independent association between LEE grade at admission (t0) and ICU length of stay (LOS), adjusted for age. Coefficients (β), standard errors, t values and p-values are reported. Positive β coefficients indicate longer LOS.

Predictor	β coefficient	Std. Error	95% CI (approx.)	p-value	% change in LOS	Interpretation
**Age (per year)**	−0.007305	0.003678	−0.0145 to −0.0001	0.0485	**−0.7%**	Older survivors had slightly shorter LOS
**LEE grade at admission (t0)**	+0.169688	0.071692	+0.029 to +0.309	0.0190	**+18.5%**	Each 1-grade ↑ LEE t0 → longer LOS
**Intercept**	2.941760	0.221131	—	<2e-16	—	—

Supplementary Table S23. Multivariable analysis restricted to ICU survivors and excluding patients with hypoxic–ischemic hepatitis (HH), evaluating whether the maximum LEE grade reached within the first 72 hours (peak LEE max) is independently associated with ICU length of stay (LOS) after adjustment for age. Regression coefficients

(β), approximate 95% confidence intervals (CI), p-values, and percent change in LOS are reported. Positive β coefficients indicate longer LOS.

Predictor	β coefficient	Std. Error	95% CI (approx.)	p-value	% change in LOS	Interpretation
**Age (per year)**	−0.007268	0.003716	−0.0146 to +0.0001	0.052	−0.7%	Borderline shorter LOS with age
**Peak LEE grade (max 72h)**	+0.096609	0.060412	−0.022 to +0.215	0.111	**+10.1%** (NS)	Trend toward longer LOS, not significant
**Intercept**	2.956597	0.227772	—	<2e-16	—	—

Supplementary Table S24. Model evaluating the independent association of log-transformed AST and ALT at ICU admission with ICU length of stay (LOS) among survivors. Regression coefficients (β), approximate 95% confidence intervals, p-values, and percentage change in LOS (exp(β) − 1) are reported. Positive values indicate longer LOS.

Predictor	β estimate	95% CI (approx.)	p-value	% change in LOS	95% CI (%)	Interpretation
**Age (per year)**	−0.00680	−0.0140 to +0.0005	0.0619	−0.68%	−1.39% to +0.05%	Borderline shorter LOS with age
**log(AST t0)**	+0.28272	+0.014 to +0.552	0.0398	**+32.6%**	+1.4% to +73.7%	Significant association
**log(ALT t0)**	−0.07706	−0.348 to +0.194	0.5751	−7.4%	−29.4% to +21.4%	Not associated with LOS

Supplementary Table S25. Sensitivity analysis restricted to ICU survivors: multivariable log-linear regression assessing the association of peak AST and ALT within 72 hours with ICU length of stay (LOS), adjusted for age. Regression coefficients (β), 95% confidence intervals, and percent change in LOS are reported. Positive β coefficients represent longer ICU LOS. Neither AST nor ALT peak values were independently associated with LOS.

Predictor	β	95% CI	p-value	% change in LOS	95% CI (%)
**Age (per year)**	−0.00665	−0.0139 to +0.00055	0.0699	–0.7%	–1.4% to +0.05%
**log(AST max 72h)**	+0.166	−0.083 to +0.414	0.190	+18.1%	–8.0% to +51.3%
**log(ALT max 72h)**	+0.0036	−0.249 to +0.257	0.977	+0.36%	–22.1% to +29.2%

Supplementary Table S26. Univariate log-linear regression analyses evaluating the association between clinical variables and duration of invasive mechanical ventilation among ICU survivors. Positive β coefficients indicate longer ventilation duration.

Variable	β	95% CI	p-value	% change	95% CI (%)
**Obesity**	0.421	0.145 to 0.697	**0.0030**	**+52.4%**	+15.6% to +100.8%
**BMI (per unit)**	0.0406	0.0091 to 0.0722	**0.0120**	**+4.15%**	+0.9% to +7.5%
**LEE grade at admission (t0)**	0.188	−0.0067 to 0.382	0.058	+20.6%	−0.7% to +46.5%
**HH (hypoxic–ischemic hepatitis)**	0.841	−0.179 to 1.86	0.105	+132%	−16.4% to +547%

Variable	β	95% CI	p-value	% change	95% CI (%)
**Diabetes**	0.491	−0.141 to 1.12	0.127	+63.5%	−13.2% to +206%
**Direct bilirubin**	−0.210	−0.517 to 0.0967	0.178	−19.0%	−40.3% to +10.1%
**Creatinine**	0.137	−0.0679 to 0.342	0.188	+14.7%	−6.6% to +40.8%
**AAR**	0.197	−0.103 to 0.498	0.197	+21.8%	−9.8% to +64.6%
**LEE grade max**	0.109	−0.0648 to 0.284	0.217	+11.6%	−6.3% to +32.8%
**Hypertension**	0.282	−0.191 to 0.756	0.241	+32.6%	−17.4% to +112%
**Age (years)**	−0.00686	−0.0199 to 0.00615	0.299	−0.68%	−1.97% to +0.62%
**Charlson comorbidity index**	−0.0512	−0.162 to 0.0601	0.365	−4.99%	−15.0% to +6.2%
**Chronic liver disease**	−0.415	−1.37 to 0.545	0.394	−33.9%	−74.1% to +72.5%
**ALT max**	0.000211	−0.000281 to 0.000704	0.398	+0.02%	−0.03% to +0.07%
**ALT t0**	0.000196	−0.000316 to 0.000708	0.451	+0.02%	−0.03% to +0.07%
**Sodium**	−0.0148	−0.0539 to 0.0243	0.455	−1.47%	−5.25% to +2.46%
**Albumin**	−0.130	−0.479 to 0.220	0.464	−12.2%	−38.1% to +24.6%

Variable	β	95% CI	p-value	% change	95% CI (%)
**INR**	−0.00752	−0.0282 to 0.0132	0.474	−0.75%	−2.8% to +1.3%
**AST max**	0.000107	−0.000191 to 0.000405	0.480	+0.01%	−0.02% to +0.04%
**AST t0**	0.000106	−0.000205 to 0.000417	0.503	+0.01%	−0.02% to +0.04%
**Chronic heart failure**	0.195	−0.428 to 0.819	0.537	+21.6%	−34.9% to +127%
**COPD**	−0.160	−0.732 to 0.413	0.582	−14.8%	−51.2% to +51.1%
**Admission diagnosis**	0.0208	−0.0553 to 0.0969	0.589	+2.1%	−5.4% to +10.2%
**Total bilirubin**	−0.0612	−0.328 to 0.206	0.651	−5.9%	−27.9% to +22.9%
**GGT**	−0.000520	−0.00328 to 0.00224	0.710	−0.05%	−0.33% to +0.22%
**ALBI score**	0.0532	−0.256 to 0.362	0.734	+5.46%	−22.7% to +43.6%
**Chronic renal failure**	−0.157	−1.50 to 1.18	0.817	−14.5%	−77.6% to +224%
**Malignancy**	−0.0581	−0.741 to 0.625	0.867	−5.64%	−52.3% to +86.7%
**Sex (male)**	0.0371	−0.430 to 0.504	0.876	+3.78%	−35.0% to +65.5%
**Asthma**	0.0694	−0.955 to 1.09	0.894	+7.18%	−61.4% to +198%
**SAPS II**	−0.000953	−0.0158 to 0.0139	0.899	−0.095%	−1.6% to +1.4%

Variable	β	95% CI	p-value	% change	95% CI (%)
**Platelets**	−0.000031	−0.00222 to 0.00216	0.978	−0.003%	−0.22% to +0.22%

Supplementary Table S27. Multivariable log-linear regression model evaluating the independent association of obesity class and LEE grade at admission with the duration of invasive mechanical ventilation among ICU survivors. Positive coefficients indicate longer ventilation time. Obesity and LEE grade at admission were both independently associated with prolonged ventilation.

Predictor	β	p-value	% change	95% CI (%)
**Obesity (per class)**	**0.432**	**0.002**	**+54%**	+17% to +102%
**LEE grade t0 (per grade)**	**0.200**	**0.039**	**+22%**	+1% to +48%

Supplementary Table S28. Multivariable log-linear regression model assessing BMI (continuous) and LEE grade at admission as predictors of invasive mechanical ventilation duration among ICU survivors. BMI remained significantly associated with longer ventilation duration, whereas the association with LEE grade at admission did not reach statistical significance.

Predictor	β	p-value	% change	95% CI (%)
**BMI (per unit)**	**0.040–0.042**	**0.010–0.012**	**+4%**	+1% to +7%
**LEE grade t0 (per grade)**	~0.17–0.19	0.055–0.11	+18–21%	includes 0

Supplementary Table S29. Multivariable log-linear regression model evaluating the association of obesity class and peak LEE grade within the first 72 hours with the duration of invasive mechanical ventilation among ICU survivors. Obesity remained a strong

independent predictor, while peak LEE grade did not demonstrate a significant association.

Predictor	β	p-value	% change	95% CI (%)
**Obesity (per class)**	**0.437**	**0.002**	**+55%**	+17% to +104%
LEE grade max (per grade)	0.130	0.133	+14%	−4% to +35%

Supplementary Table S30. Multivariable log-linear regression model assessing BMI (continuous) and peak LEE grade as predictors of invasive mechanical ventilation duration among ICU survivors. BMI remained significantly associated with ventilation duration, whereas peak LEE grade did not show an independent effect.

Predictor	β	p-value	% change	95% CI (%)
**BMI (per unit)**	**0.041**	**0.011**	**+4.2%**	+1% to +7.5%
LEE grade max (per grade)	0.115	0.187	+12%	−6% to +33%
